# Supplementary material for: Maternal intrahepatic cholestasis of pregnancy and neurodevelopmental conditions in offspring: A population-based cohort study of 2 million Swedish children
Source: PLoS Med. 2024 Jan 16;21(1):e1004331. doi: 10.1371/journal.pmed.1004331 (PMC10790993; doi:10.1371/journal.pmed.1004331)
Supplement: S2 Appendix — Prospectively recorded analysis plan. (DOCX) [file pmed.1004331.s002.docx]

| **Department of Global Public Health**  Epidemiology of Psychiatric Conditions, Substance use and Social Environment (EPiCSS) |  |
| --- | --- |

# Psychiatry Sweden Linkage(PS) Research Description Form(RDF)

# for projects in the research group EPiCSS

# A) Contact information

**Date of submission:** 18/12/2021

**Title of project [Abbreviation]:** Maternal intrahepatic cholestasis of pregnancy and neurodevelopmental disorders in offspring.

**Principal investigator for the project**: Renee Gardner (main supervisor)

**Principal investigator affiliated to KI (Yes/No):** Yes

**Names and affiliations of other investigators (if any):** Shuyun Chen (PhD student), Viktor H. Ahlqvist (PhD student)

**Names of all persons who need access to the datasets to be prepared:** Renee Gardner, Shuyun Chen (PhD student), Viktor H. Ahlqvist (PhD student)

**Address:** Institutionen för global folkhälsa, Karolinska Institutet, SE-171 77 Stockholm

**E-mail:** [renee.gardner@ki.se](mailto:renee.gardner@ki.se), [shuyun.chen@ki.se](mailto:shuyun.chen@ki.se), [viktor.ahlqvist@ki.se](mailto:viktor.ahlqvist@ki.se)

# B) Description of the project

## Background (short):

Intrahepatic cholestasis of pregnancy (ICP) is a metabolic disease unique to pregnancy with high recurrence rates in singleton pregnancies and usually resolves rapidly after delivery. The average incidence of ICP is 0.1–2% with a large variance over countries and population. It is characterized by unexplained pruritus, elevated serum bile acid and/or transaminases in the late second and third trimester of pregnancy. Though the aetiology of ICP is not fully understood, it is likely caused by a combination of genetic, hormonal, and environmental factors. ICP increases the risk of stillbirth, preterm birth, meconium-stained amniotic fluid, and admission to the neonatal unit. Interestingly, ursodeoxycholic acid (UDCA) has recently been proposed as a viable treatment for ICP. There are, however, some concerns that ursodeoxycholic acid does not improve fetal outcomes.

Unfortunately, little is known about the role of maternal ICP in offspring neurodevelopmental health. This is especially concerning as consequences of ICP include established determinants of offspring neurodevelopment. For example, it is not unreasonable to expect that ICP has a causal effect on offspring neurodevelopmental disorders (NDDs) if ICP has a causal effect on prematurity (regardless of whether it is iatrogenic or spontaneous) and prematurity has a causal effect on offspring NDDs. Furthermore, there are suggestions that maternal hyperglycemia/adiposity-induced inflammation and increased serum leptin levels influence fetal neurodevelopment. Offspring of cholic acid-fed mice were also found to have elevated levels of serum leptin and pro-inflammatory markers (e.g., TNF-α and CRP). Additionally, a higher level of maternal bile acid is related to placental vessel spasm and potentially subsequent impairment of fetal perfusion and oxygenation. There are also suggestions that cholic acid can pass the placenta and affect the foetus directly. Animal studies showed various bile acid transporters are expressed in the central nervous system (CNS), such as in the hypothalamus and frontal cortex. These findings suggest that bile acid signalling in the CNS may be implicated in neurodegenerative diseases.

Yet, no previous study has examined the effect of maternal ICP on the risk of NDDs in offspring, such as autism spectrum disorders (ASD), attention deficit hyperactivity disorder (ADHD) and intellectual disability (ID) – limiting the current appreciation for the consequences of maternal ICP.

## Aims of the assignment:

To determine the role of maternal ICP in offspring NDDs.

## Specific research questions:

1. What are the associations between maternal ICP and offspring NDDs?
2. Is the association between maternal ICP and NDDs explained by shared familial factors (i.e., family-level confounders)?
3. Do the associations between maternal ICP and NDDs in offspring vary by the timing of ICP diagnosis?
   1. It is not unreasonable to believe that there exists a critical period where the effect of elevated serum bile acid is more prominent (i.e., the period of fetal neurological development). Similarly, it is not unreasonable to believe that this effect would be dose-dependent - the effect would be different depending on the cumulative exposure to serum bile acid (e.g., 3 weeks of “exposure”).
      1. These two distinct hypotheses will be tested by 1) studying the debuting time of ICP, 2) studying the length of fetal ICP exposure during gestation.
4. Does the association between ICP and offspring NDD vary by maternal treatment of ursodeoxycholic acid (ATC: A05AA02)?

***Feedback from the statistician in the group (H. Sjöqvist) (2022-02-11):*** *After reviewing the Prescription Drug Register (2005-), we observed that the utilization of UDCA for treating ICP was rare, which limited the information available for our analyses. This could be due to the fact that the use of UDCA for ICP treatment was not widespread in Sweden during that period. Consequently, we did not incorporate this research aim in the final paper, and we note this as a limitation of the work.*

# C) A detailed description of the population

## Study population

We will include singleton children born in Sweden between 1973 and 2010 (as per their availability in MBR), which we will follow till 2016 in the national patient registry. Children born outside Sweden, or with missing personnummer will be excluded - as they are not informative for our study. Any woman delivering in Sweden is eligible for enrolment. We might, however, restrict the cohort to deliveries after 1987 if we deem the ICP diagnosis under ICD-8 unreliable. We will need information on family relations (i.e., family id variable or biological maternal and biological paternal lopnr), to facilitate full-sibling analysis.

We might include further restrictions, such as excluding children residing in Sweden for less than 5 years or excluding children with co-occurrence of congenital malformations and NDDs, but we will not need these to be filtered out initially by data managers of PS. ***Revision (2022-03-04):*** *Given that most of the variables we adjusted for—including child’s sex, birth year, birth order, maternal psychiatric history, and birth month—had no missing values, and the missing values in a few adjusted covariates (i.e., maternal age, highest parental education level, and maternal birth country) were minimal (N_missing_=1697 [0.1%]), we opted to exclude those with missing values in these variables.*

## Exposure

We will identify ICP cases by ICD-8: 639.00, 639.01, 639.09, ICD-9: 646.7 and ICD-10: O26.6, from MBR and NPR, using the date of the first diagnosis as a proxy for date of onset. We will only analyse time of onset in the sub-population which have their diagnosis recorded in NPR – as there is no timing information in the MBR. We will also need information on ursodeoxycholic acid [A05AA02], both as a marker of severity and as a potential effect-modifier. We wish to have information on ursodeoxycholic acid from both MBR (1995-2010) and the drug dispensation registry (2005-).

## Follow-up/outcomes

ASD, ADHD and ID cases will be identified by codes as following: ASD (ICD-8: 299.00, 299.01, 299.02, 299.03; ICD-9: 299; ICD-10: F84); ADHD (ICD-8: 308.3; ICD-9: 314, ICD-10: F90, Prescription Drug Register: methylphenidate [N06BA04] or atomoxetine [N06BA09]); ID (ICD-8: 311–315, ICD-9: 317-319, ICD-10: F70-F79). Potential confounders include children’s sex, birth year, birth month, maternal age, parity, maternal BMI, maternal birth country, highest parental education level, and maternal psychiatric history.

## Statistical analysis

We will use logistic regression to assess the associations between ICP and NDDs in offspring. Furthermore, we will assess the association between ICP diagnosed at different times during pregnancy (i.e., <30, 30-<34 and ≥34 weeks of gestational age) and NDDs in offspring. We will also conduct sibling analysis, using conditional logistic regression, to address potential confounding from shared familial factors (i.e., genetic and environmental factors). We may resort to the between-within modelling framework to increase efficiency in sibling analysis. All standard errors will be computed using the robust (sandwich) method, to account for the correlation between siblings.

***Revision 1 (2022-03-08):*** *After discussions with our collaborators, we have revised the categorization of ICP based on trimesters to simplify the interpretation of our results. The new categories are: <28 weeks, 28-36 weeks, and ≥37 weeks of gestation.*

***Revision 2 (2023-09-28):*** *After reviewing the feedback from the editor and reviewers of PLOS Medicine, we conducted additional analyses: a) Crude model without any adjustments; b) Adjusting for GA as a linear term, as categories of gestational weeks, and using restricted cubic splines; c) We performed multiple imputation and IPW to deal with missing data in maternal BMI.*

# D) List of variables from Psychaiatry Sweden (PS)

## Exposures, outcomes and covariates

| **Variable** | **Variable options** |
| --- | --- |
| **Exposure** |  |
| ICP | Binary  0 “No” 1 “Yes” |
| ICP diagnostic date | date |
| **Outcomes** |  |
| Any ASD | 0 “Without ASD, ID or ADHD” 1 “with ASD” |
| Any ID | 0 “Without ASD, ID or ADHD” 1 “with ID” |
| Any ADHD | 0 “Without ASD, ID or ADHD” 1 “with ADHD” |
| ASD date | Date of first ASD diagnosis |
| ID date | Date of first ID diagnosis |
| ADHD | Date of first ADHD diagnosis |
| **Covariates** |  |
| Sex | 0 “Female” 1 “Male” |
| Maternal age | Continuous |
|  | Categorical  1 “<25”  2 “25-29”  3 “30-34”  4 “35-39”  5 “≥40” |
| Birth year | Continuous (Per year) |
|  | Categorical  1 “1987-1992”  2 “1993-1998”  3 “1999-2004”  4 “2005-2010” |
| Birth month | Continuous (1-12) |
|  | Categorical  1 “January-March”  2 “April-June”  3 “July-September”  4 “October-December” |
| Birth order | Categorical  1 “1”  2 “2”  3 “≥3” |
| Maternal BMI at first antenatal care | Continuous |
|  | Categorical  0 “Normal weight (18.5-24.9 kg/m2)”  1 “Underweight (<18.5 kg/m2)”  2 “Overweight (25.0 – 29.9 kg/m2)”  3 “Obese (≥30 kg/m2)” |
| Maternal birth country region | Categorical  1 “Nordic”  2 “Europe”  3 “Africa”  4 “Asia”  5 “Other” |
| The highest income level of the parents | Categorical  1=lowest, 2, 3, 4, 5=highest |
| Maternal psychiatric history | Binary  0 “No” 1 “Yes” |
| Offspring emigration | Emigration date |
| Offspring immigration date | Immigration date |
| **Other obstetric and neonatal covariates** |  |
| Gestational hypertensive conditions | Binary  0 “No” 1 “Yes” |
| Gestational diabetes mellitus | Binary  0 “No” 1 “Yes” |
| Birth weight for gestational age | Categorical  0 “Appropriate for gestational age”  1 “Small for gestational age”  2 “Large for gestational age” |
| Mode of delivery | Categorical  0 “Vaginal non-instrumental”  1 “Vaginal instrumental”  2 “Cesarean section” |
| Induction of labor | Categorical  0 “Spontaneous onset”  1 “Induced Labor”  2 “Cesarean section before labor onset” |
| Gestational age at birth | Categorical  0 “Term (37-<42 weeks)”  1 “Preterm (<37 weeks)”  2 “Post-term (≥42 weeks)” |
|  | Continuous  Gestational weeks at birth |
|  | Continuous  Gestational days at birth |
| Apgar score at 5-minute | Binary  0 “≥7”  1 “<7” |
| Neonatal asphyxia-related comorbidities | Binary  0 “No” 1 “Yes” |
| Neonatal hypoglycemia | Binary  0 “No” 1 “Yes” |
| Neonatal jaundice | Binary  0 “No” 1 “Yes” |

## E) List of ICD codes for diagnoses (outcomes, exposures, confounders etc)

***Notes:*** *This protocol builds upon other studies that were carried out within the research group.*

All the putative mediating obstetric variables (GDM, preeclampsia/eclampsia, delivery mode, intrauterine hypoxia) and neonatal variables (gestational age at birth, size for gestational age, Apgar score at 5-minute, neonatal asphyxia-related comorbidities) are in the dataset. (For ICD codes, see the application form for Shuyun’s mediation protocol [*Associations between maternal metabolic conditions and neurodevelopmental conditions in offspring: the mediating effects of obstetric and neonatal complications (BMC Medicine in press 2023) Shuyun Chen; Xi Wang; Brian K. Lee; Renee M. Gardner])*

In extension to those of Shuyun’s mediation protocol we will be working with:

| **Description** | **ICD-8** | **ICD-9** | **ICD-10** | **ATC** |
| --- | --- | --- | --- | --- |
| ASD | 299.00, 299.01, 299.02, 299.03 | 299 | F84 |  |
| ID | 311–315 | 317-319 | F70-F79 |  |
| ADHD | 308.3 | 314 | F90 | methylphenidate [N06BA04] or atomoxetin [N06BA09] |
| Intrahepatic cholestasis of pregnancy | 639.00, 639.01, 639.09 | 646.7 | O26.6 | - |
| Ursodeoxycholic acid | - | - | - | A05AA02 |
