# Supplementary material for: Maternal intrahepatic cholestasis of pregnancy and neurodevelopmental conditions in offspring: A population-based cohort study of 2 million Swedish children
Source: PLoS Med. 2024 Jan 16;21(1):e1004331. doi: 10.1371/journal.pmed.1004331 (PMC10790993; doi:10.1371/journal.pmed.1004331)
Supplement: S1 Fig — (DOCX) [file pmed.1004331.s003.docx]

**
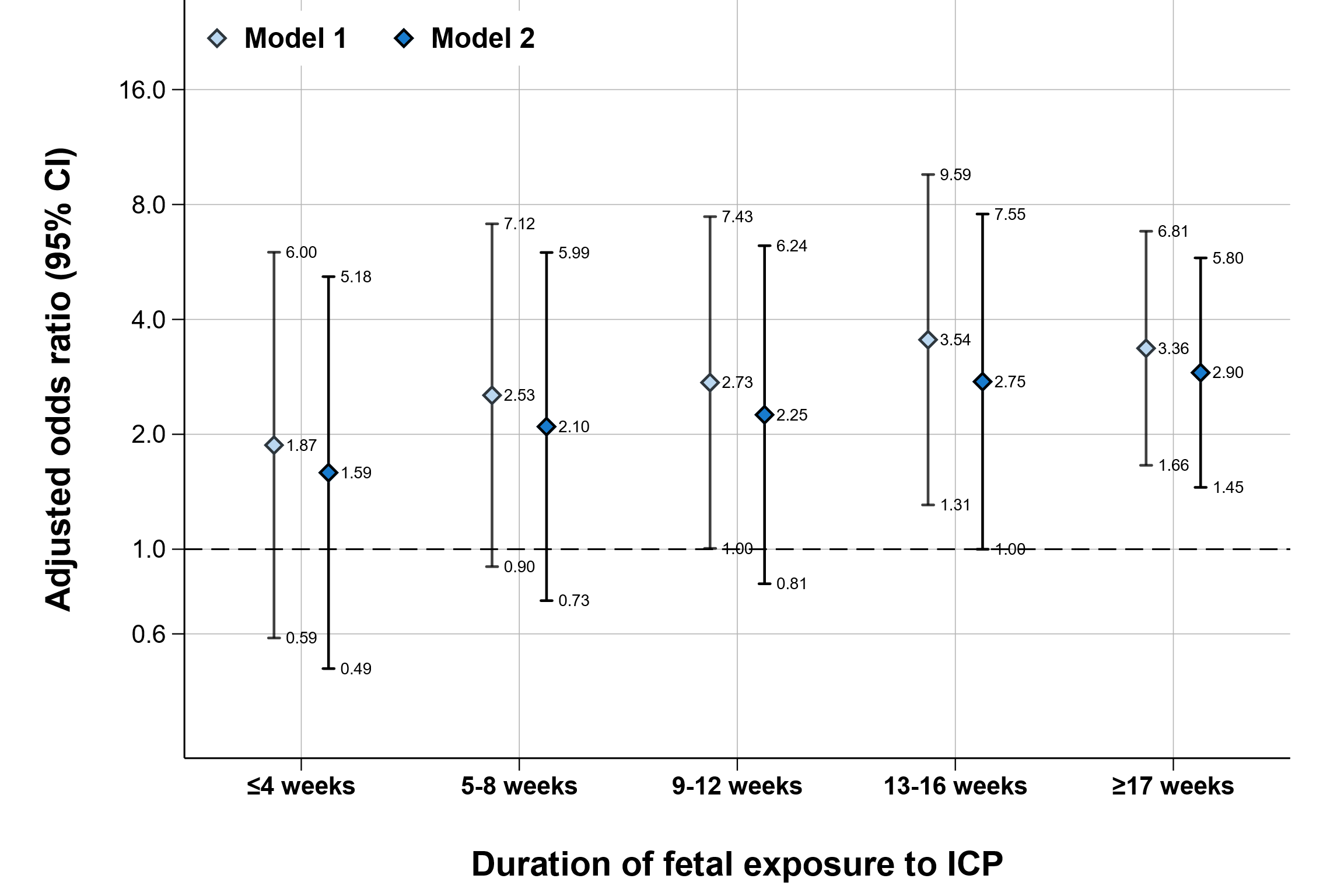
S1 Fig.** The association between duration of fetal exposure to maternal intrahepatic cholestasis and any offspring neurodevelopmental conditions, and the distribution of week of onset across duration of exposure.

|  | **Duration of fetal exposure to ICP** | | | | |
| --- | --- | --- | --- | --- | --- |
|  | **≤4 weeks** | **5-8 weeks** | **9-12 weeks** | **13-16 weeks** | **≥17 weeks** |
| **Gestational week of ICP diagnosis** |  |  |  |  |  |
| **<28** | 13 (0.2%) | 17 (1.6%) | 72 (22.7%) | 101 (95.3%) | 141 (100%) |
| **28-36** | 2,540 (28.9%) | 994 (95.8%) | 220 (75.3%) | 5 (4.7%) | 0 |
| **≥37** | 6,248 (71.0%) | 27 (2.6%) | 0 | 0 | 0 |

**Abbreviations:** ICP-Intrahepatic cholestasis of pregnancy

The referent group is the unexposed group of ICP. **Model 1:** Logistic regression models with standard errors computed using the robust (sandwich) method. Adjusted for child’s sex, birthyear, gestational age at delivery, and gestational week at ICP diagnosis. **Model 2:** Adjusted for model 1 and maternal age, highest parental education level, maternal birth country, birth order, maternal psychiatric history, and birth month.
