# Supplementary material for: Maternal intrahepatic cholestasis of pregnancy and neurodevelopmental conditions in offspring: A population-based cohort study of 2 million Swedish children
Source: PLoS Med. 2024 Jan 16;21(1):e1004331. doi: 10.1371/journal.pmed.1004331 (PMC10790993; doi:10.1371/journal.pmed.1004331)
Supplement: S2 Fig — (DOCX) [file pmed.1004331.s004.docx]

**S2 Fig.** The association between gestational week of maternal intrahepatic cholestasis diagnosis and any offspring neurodevelopmental conditions, among the offspring exposed to maternal intrahepatic cholestasis (N=10 378), separated by full-cohort analysis and full-sibling analysis.

**
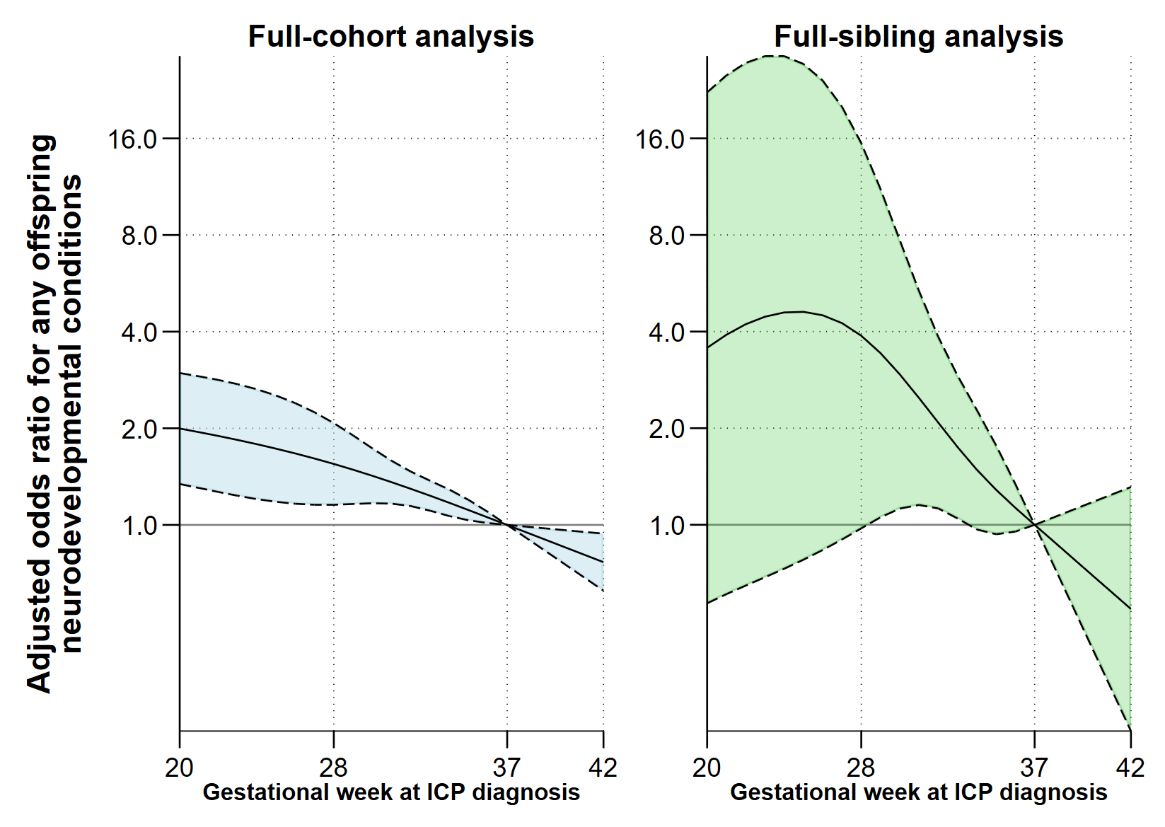
**

**Abbreviations:** ICP-Intrahepatic cholestasis of pregnancy.

All the analyses were restricted to the individuals exposed to ICP. The curved solid black line represents the odds ratio (OR) calculated through restricted cubic splines models: 4 knots placed at 14, 28, 34, and 37 weeks of gestation. A reference line is included for an OR of 1.00. Conditional logistic regression was used for the sibling analysis, with standard errors computed by robust (sandwich) method. The models were adjusted for child’s sex, birth year, maternal age, highest parental education level, maternal birth country, birth order, maternal psychiatric history, and birth month.
