# Supplementary material for: Maternal intrahepatic cholestasis of pregnancy and neurodevelopmental conditions in offspring: A population-based cohort study of 2 million Swedish children
Source: PLoS Med. 2024 Jan 16;21(1):e1004331. doi: 10.1371/journal.pmed.1004331 (PMC10790993; doi:10.1371/journal.pmed.1004331)
Supplement: S3 Fig — (DOCX) [file pmed.1004331.s005.docx]

**S3 Fig.** The association between timing at maternal intrahepatic cholestasis diagnosis, as a function of the percentage of pregnancy completed at diagnosis, and any offspring neurodevelopmental conditions among children exposed to ICP (N=10,378).

**
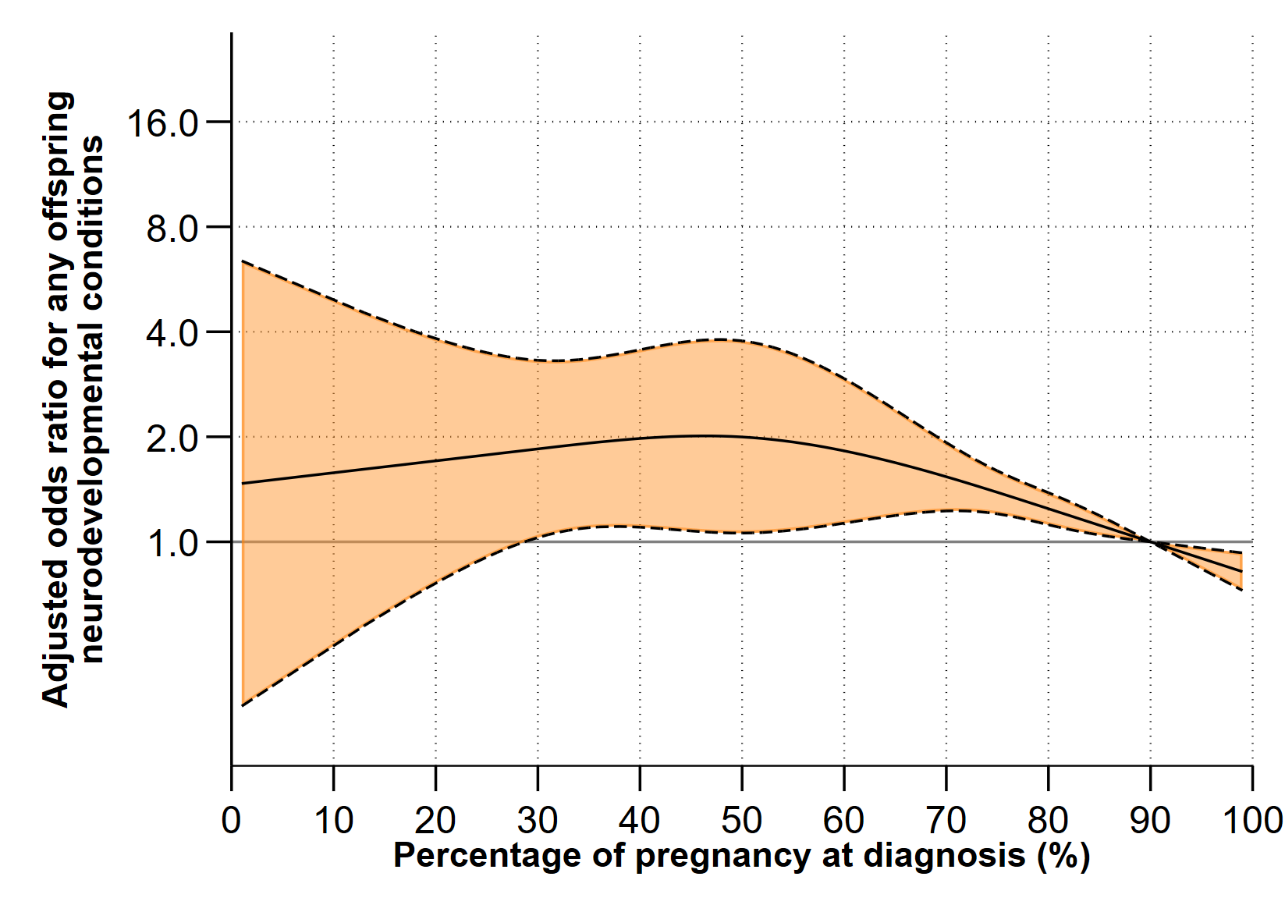
**

**Abbreviations:** ICP-Intrahepatic cholestasis of pregnancy.

The analysis was restricted to the individuals exposed to ICP (N=10 378). The percentage of pregnancy at ICP diagnosis was calculated by formula: gestational week at ICP diagnosis/total gestational week of pregnancy. The curved solid black line represents the odds ratio (OR) calculated through restricted cubic splines models: 4 knots placed at 30%, 50%, 80%, and 90 % of pregnancy. A reference line is included for an OR of 1.00. The model was adjusted for child’s sex, birth year, maternal age, highest parental education level, maternal birth country, birth order, maternal psychiatric history, and birth month.
