# Supplementary material for: Maternal intrahepatic cholestasis of pregnancy and neurodevelopmental conditions in offspring: A population-based cohort study of 2 million Swedish children
Source: PLoS Med. 2024 Jan 16;21(1):e1004331. doi: 10.1371/journal.pmed.1004331 (PMC10790993; doi:10.1371/journal.pmed.1004331)
Supplement: S4 Fig — (DOCX) [file pmed.1004331.s006.docx]

**S4 Fig.** The association between the categorical timing of intrahepatic cholestasis of pregnancy diagnosis and any neurodevelopmental conditions in offspring among those who were born to mothers with ICP (N=10,378).

**
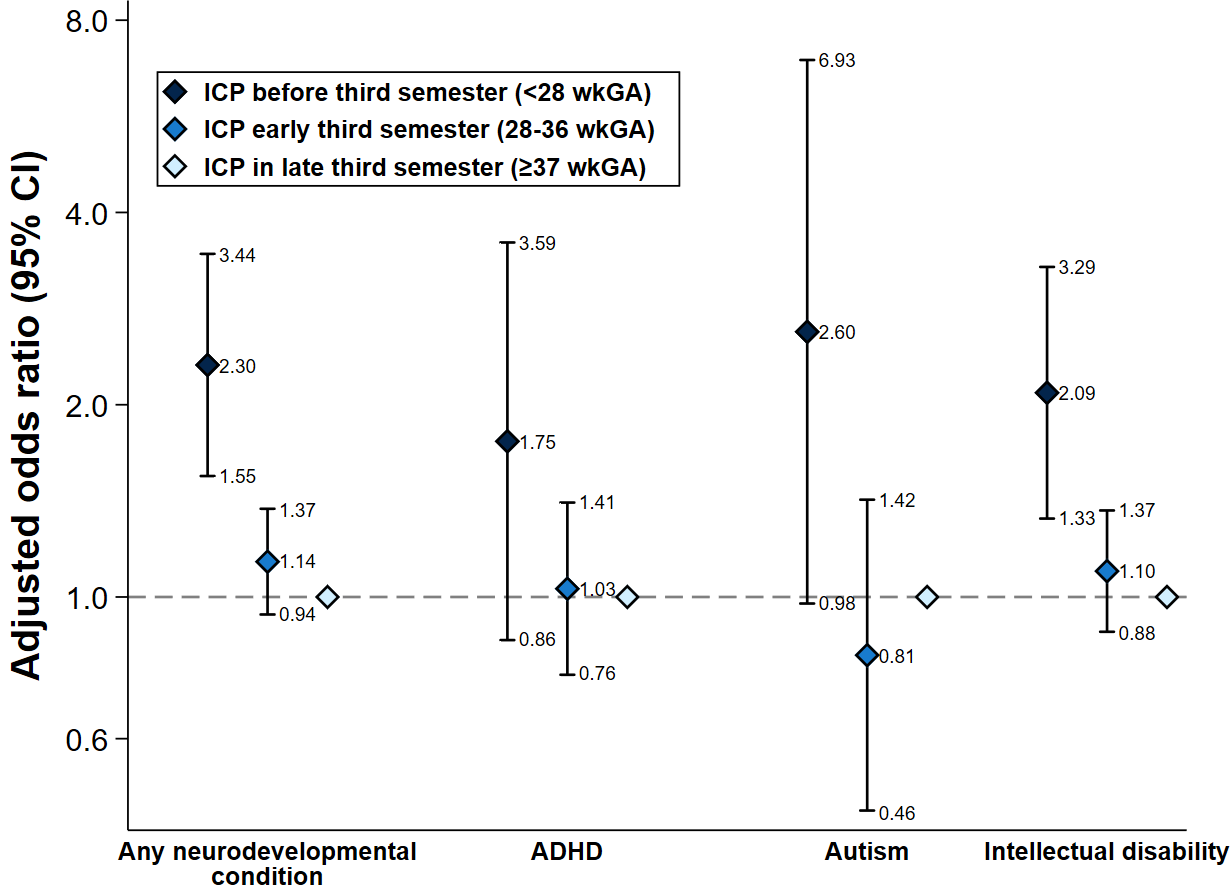
**

**Abbreviations:** ICP-Intrahepatic cholestasis of pregnancy; ADHD-Attention deficit/hyperactivity disorder; wkGA- weeks of gestational age.

All the analyses were restricted to the individuals exposed to ICP. A reference line is included for an OR of 1.00. Intrahepatic cholestasis of pregnancy diagnosed ≥37 weeks of gestation was the referent group. Logistic regression was used for the analysis, with standard errors computed by robust (sandwich) method. The models were adjusted for child’s sex, birth year, maternal age, highest parental education level, maternal birth country, birth order, maternal psychiatric history, and birth month.
