# Supplementary material for: Maternal intrahepatic cholestasis of pregnancy and neurodevelopmental conditions in offspring: A population-based cohort study of 2 million Swedish children
Source: PLoS Med. 2024 Jan 16;21(1):e1004331. doi: 10.1371/journal.pmed.1004331 (PMC10790993; doi:10.1371/journal.pmed.1004331)
Supplement: S5 Fig — (DOCX) [file pmed.1004331.s007.docx]

**S5 Fig.** Directed Acyclic Graph illustrating the path dependencies with gestational age.


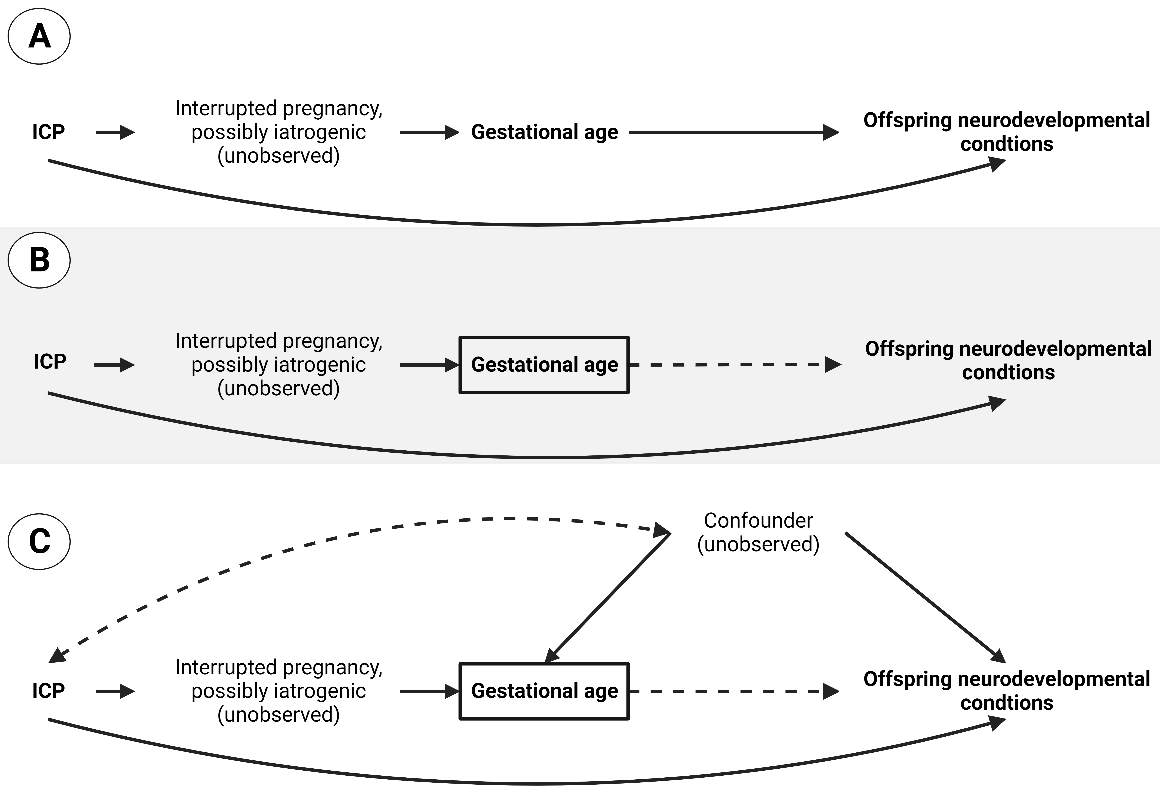


Panel (A) illustrates the scenario when we don’t adjust for gestational age. In this case, the effect of ICP on NDC is unbiased. Panel (B) depicts the scenario where we do adjust for gestational age, which obstructs an effect and biases the estimate toward the null. Panel (C) presents the scenario where there is a residual confounder between gestational age and NDCs. In this situation, gestational age acts as a collider, leading to biased effect estimates with unpredictable directions.
