# Supplementary material for: Maternal intrahepatic cholestasis of pregnancy and neurodevelopmental conditions in offspring: A population-based cohort study of 2 million Swedish children
Source: PLoS Med. 2024 Jan 16;21(1):e1004331. doi: 10.1371/journal.pmed.1004331 (PMC10790993; doi:10.1371/journal.pmed.1004331)
Supplement: S1 Table — (DOCX) [file pmed.1004331.s008.docx]

**S1 Table.** Extended details on variable definitions and their underlying ICD/ATC codes.

| **Variable** | **Underlying condition(s)** | **ICD-9^ab^** | **ICD-10^ab^** | **ICD-8^ab^** | **Anatomical Therapeutic Chemical codes** |
| --- | --- | --- | --- | --- | --- |
| Intrahepatic cholestasis of pregnancy | Intrahepatic cholestasis of pregnancy | 646H | O26.6 |  |  |
| Gestational hypertensive conditions | Pre-eclampsia/Gestational [pregnancy-induced] hypertension | 642E, 642F, 642X | O13, O14 |  |  |
|  | Eclampsia | 642G, 642H | O15 |  |  |
| Neonatal asphyxia-related comorbidities | Birth asphyxia | 768F, 768G, 768X, 770W | P21 |  |  |
|  | Neonatal aspiration syndromes | 770B | P24 |  |  |
|  | Respiratory distress of new-born | 769X,770G,770W | P22 |  |  |
|  | Convulsions | 779A | P90 |  |  |
|  | Hypoxic ischemic encephalopathy and related conditions | 779B, 779C | P910, P913-P916, P918, P919 |  |  |
| Neonatal hypoglycaemia | | 775G | P703, P704 |  |  |
| Gestational diabetes mellitus (GDM) | | 648W | O24.4 |  |  |
| Autism | | 299 | F84 |  |  |
| Attention deficit/hyperactivity disorder (ADHD) | | 314 | F90 |  | methylphenidate [N06BA04] or atomoxetin [N06BA09] |
| Intellectual disability | | 317-319 | F70-F79 |  |  |
| Maternal psychiatric history | | 290-319 | F chapter | 290-315 |  |
| **Variable** | **Underlying variable** | **Calculation** | | | |
| Small for gestational age | Z-score <-2 | Male: Mean (birthweight) = (-(1.907345*10^(-6))*days of gestation^4 + (1.140644*10^(-3))*days of gestation^3 -0.1336265*days^2 +1.976961*days of gestation+241.0053)  Female: Mean (birthweight)= (-(2.761948*10^(-6))* days of gestation ^4 + (1.744841*10^(-3))* days of gestation s^3 -0.2893623* days of gestation^2 + 18.91197* days of gestation-413.5122)  Z-score= (birthweight – mean [birthweight])/(0.12 * mean [birthweight]) | | | |
| Large for gestational age | Z-score > 2 | As above | | | |
| Preterm birth | Gestational week at birth<37 weeks | Based on gestational week either using ultrasound or last menstrual period. | | | |

^a^The National Patient Register (NPR): including inpatient care beginning in 1973, outpatient physician visits in specialist care beginning in 1997, and outpatient psychiatric diagnoses from 2006, children and adolescent psychiatric care (2011)

^b^The Medical Birth Register (MBR): including information from medical records from prenatal, delivery, and neonatal healthcare beginning in 1973.

^c^The Prescribed Drug Register (PDR) contains data on Anatomical Therapeutic Chemical (ATC) classification codes for medications dispensed to the entire population in Sweden since 1 July 2005. Receipt of a prescription for attention-deficit/hyperactivity disorder (ADHD) medications is a useful proxy for an ADHD diagnosis, as Swedish medical guidelines mandate that ADHD medications should only be prescribed by a psychiatric specialist and after other (non-pharmacological) interventions have failed.
