# Supplementary material for: Maternal intrahepatic cholestasis of pregnancy and neurodevelopmental conditions in offspring: A population-based cohort study of 2 million Swedish children
Source: PLoS Med. 2024 Jan 16;21(1):e1004331. doi: 10.1371/journal.pmed.1004331 (PMC10790993; doi:10.1371/journal.pmed.1004331)
Supplement: S2 Table — (DOCX) [file pmed.1004331.s009.docx]

**S2 Table.** Characteristics of the study sample over diagnoses of neurodevelopmental conditions in offspring.

|  | **Unaffected** | **Any neurodevelopmental conditions** | **ADHD** | **Autism** | **Intellectual disability** | |
| --- | --- | --- | --- | --- | --- | --- |
| **Total** | 2,232,110 | 143,746 | 106,381 | 48,363 | 23,933 | |
| **Sex** |  |  |  |  |  | |
| Female | 1,103,218 (49.4%) | 51,455 (35.8%) | 37,605 (35.3%) | 15,237 (31.5%) | 9,569 (40.0%) | |
| Male | 1128892 (50.6%) | 92,291 (64.2%) | 68,776 (64.7%) | 33,126 (68.5%) | 14,364 (60.0%) | |
| **Birthyear** |  |  |  |  |  | |
| 1987-1992 | 631,576 (28.3%) | 38,034 (26.5%) | 26,656 (25.1%) | 12,308 (25.4%) | 7,470 (31.2%) | |
| 1993-1998 | 523,751 (23.5%) | 45,918 (31.9%) | 34,829 (32.7%) | 14,780 (30.6%) | 7,522 (31.4%) | |
| 1999-2004 | 489,064 (21.9%) | 39,962 (27.8%) | 31,366 (29.5%) | 13,461 (27.8%) | 5,394 (22.5%) | |
| 2005-2010 | 587,719 (26.3%) | 19,832 (13.8%) | 13,530 (12.7%) | 7,814 (16.2%) | 3,547 (14.8%) | |
| **Birth months** |  |  |  |  |  | |
| January-March | 573,821 (25.7%) | 33,739 (23.5%) | 24,501 (23.0%) | 11,640 (24.1%) | 5,922 (24.7%) | |
| April-June | 598,751 (26.8%) | 36,412 (25.3%) | 26,738 (25.1%) | 12,348 (25.5%) | 6,191 (25.9%) | |
| July-September | 571,424 (25.6%) | 37,857 (26.3%) | 28,188 (26.5%) | 12,605 (26.1%) | 6,211 (26.0%) | |
| October-December | 488,114 (21.9%) | 35,738 (24.9%) | 26,954 (25.3%) | 11,770 (24.3%) | 5,609 (23.4%) | |
| **Maternal age** |  |  |  |  |  | |
| <25 | 412,056 (18.5%) | 37,922 (26.4%) | 29,991 (28.2%) | 11,010 (22.8%) | 5,915 (24.7%) | |
| 25-29 | 759,452 (34.0%) | 47,099 (32.8%) | 35,131 (33.0%) | 15,676 (32.4%) | 7,598 (31.7%) | |
| 30-34 | 697,609 (31.3%) | 37,659 (26.2%) | 26,988 (25.4%) | 13,428 (27.8%) | 6,362 (26.6%) | |
| 35-39 | 303,854 (13.6%) | 17,291 (12.0%) | 11,837 (11.1%) | 6,677 (13.8%) | 3,231 (13.5%) | |
| ≥40 | 59,139 (2.6%) | 3,775 (2.6%) | 2,434 (2.3%) | 1,572 (3.3%) | 827 (3.5%) | |
| **Highest parental education level** | |  |  |  | |  |
| Primary school | 70,855 (3.2%) | 7,589 (5.3%) | 5,486 (5.2%) | 1,958 (4.0%) | 2,118 (8.8%) | |
| Upper secondary school | 912,831 (40.9%) | 75,511 (52.5%) | 57,959 (54.5%) | 22,435 (46.4%) | 13,433 (56.1%) | |
| University level | 1,248,424 (55.9%) | 60,646 (42.2%) | 42,936 (40.4%) | 23,970 (49.6%) | 8,382 (35.0%) | |
| **Maternal birth country** | |  |  |  | |  |
| Nordic | 1,916,773 (85.9%) | 127,891 (89.0%) | 96,533 (90.7%) | 42,573 (88.0%) | 19,553 (81.7%) | |
| Europe | 96,634 (4.3%) | 4,286 (3.0%) | 2,637 (2.5%) | 1,686 (3.5%) | 1,074 (4.5%) | |
| Africa | 41,577 (1.9%) | 2,235 (1.6%) | 1,346 (1.3%) | 791 (1.6%) | 783 (3.3%) | |
| Asia | 148,318 (6.6%) | 6,950 (4.8%) | 4,174 (3.9%) | 2,454 (5.1%) | 2,146 (9.0%) | |
| Other | 28,808 (1.3%) | 2,384 (1.7%) | 1,691 (1.6%) | 859 (1.8%) | 377 (1.6%) | |
| **Maternal psychiatric history** | 107,347 (4.8%) | 12,904 (9.0%) | 9,833 (9.2%) | 4,511 (9.3%) | 2,156 (9.0%) | |
| **Birth order** |  |  |  |  |  | |
| 1 | 956,209 (42.8%) | 62,725 (43.6%) | 46,304 (43.5%) | 22,527 (46.6%) | 9,427 (39.4%) | |
| 2 | 811,767 (36.4%) | 48,159 (33.5%) | 36,004 (33.8%) | 15,407 (31.9%) | 8,089 (33.8%) | |
| ≥3 | 464,134 (20.8%) | 32,862 (22.9%) | 24,073 (22.6%) | 10,429 (21.6%) | 6,417 (26.8%) | |
| **Early-pregnancy Maternal BMI^a^** | |  |  |  | |  |
| Underweight (<18.5 kg/m^2^) | 54,119 (2.4%) | 3,670 (2.6%) | 2,683 (2.5%) | 1,248 (2.6%) | 670 (2.8%) | |
| Normal weight (18.5-24.9 kg/m^2^) | 1,104,271 (49.5%) | 61,655 (42.9%) | 45,920 (43.2%) | 20,914 (43.2%) | 9,284 (38.8%) | |
| Overweight (25.0 – 29.9 kg/m^2^) | 374,385 (16.8%) | 25,701 (17.9%) | 19,248 (18.1%) | 8,727 (18.0%) | 4,334 (18.1%) | |
| Obese (≥30 kg/m^2^) | 144,225 (6.5%) | 14,186 (9.9%) | 10,742 (10.1%) | 4,806 (9.9%) | 2,584 (10.8%) | |
| Missing | 555,110 (24.9%) | 38,534 (26.8%) | 27,788 (26.1%) | 12,668 (26.2%) | 7,061 (29.5%) | |
| **Gestational hypertensive conditions^b^** | 93,701 (4.2%) | 7,502 (5.2%) | 5,340 (5.0%) | 2,729 (5.6%) | 1,448 (6.1%) | |
| **Gestational diabetes mellitus** | 19,780 (0.9%) | 1,610 (1.1%) | 1,083 (1.0%) | 603 (1.2%) | 350 (1.5%) | |
| **Birth weight for gestational age** | |  |  |  | |  |
| Appropriate for gestational age | 2,091,948 (93.7%) | 131,274 (91.3%) | 98,002 (92.1%) | 44,051 (91.1%) | 20,678 (86.4%) | |
| Small for gestational age | 50,957 (2.3%) | 5,773 (4.0%) | 3,594 (3.4%) | 1,914 (4.0%) | 1,989 (8.3%) | |
| Large for gestational age | 77,458 (3.5%) | 5,496 (3.8%) | 4,023 (3.8%) | 2,004 (4.1%) | 919 (3.8%) | |
| Missing | 11,747 (0.5%) | 1,203 (0.8%) | 762 (0.7%) | 394 (0.8%) | 347 (1.4%) | |
| **Mode of delivery** |  |  |  |  |  | |
| Vaginal non-instrumental | 1,782,794 (79.9%) | 111,070 (77.3%) | 83,117 (78.1%) | 36,743 (76.0%) | 17,664 (73.8%) | |
| Vaginal instrumental | 153,768 (6.9%) | 9,811 (6.8%) | 7,200 (6.8%) | 3,545 (7.3%) | 1,460 (6.1%) | |
| Cesarean section | 295,548 (13.2%) | 22,865 (15.9%) | 16,064 (15.1%) | 8,075 (16.7%) | 4,809 (20.1%) | |
| **Induction of labor** |  |  |  |  |  | |
| Spontaneous onset | 1,524,863 (68.3%) | 97,448 (67.8%) | 73,465 (69.1%) | 32,645 (67.5%) | 15,024 (62.8%) | |
| Induced Labor | 174,354 (7.8%) | 13,741 (9.6%) | 10,189 (9.6%) | 4,886 (10.1%) | 2,399 (10.0%) | |
| Cesarean section before labor onset | 120,811 (5.4%) | 9,484 (6.6%) | 6,724 (6.3%) | 3,401 (7.0%) | 2,004 (8.4%) | |
| Missing | 412,082 (18.5%) | 23,073 (16.1%) | 16,003 (15.0%) | 7,431 (15.4%) | 4,506 (18.8%) | |
| **Gestational age at birth** | |  |  |  | |  |
| Preterm (<37 weeks) | 107,708 (4.8%) | 10,380 (7.2%) | 6,936 (6.5%) | 3,466 (7.2%) | 2,855 (11.9%) | |
| Term (37-<42 weeks) | 1,962,465 (87.9%) | 122,546 (85.3%) | 91,682 (86.2%) | 41,043 (84.9%) | 19,261 (80.5%) | |
| Post-term (≥42 weeks) | 159,556 (7.1%) | 10,578 (7.4%) | 7,603 (7.1%) | 3,790 (7.8%) | 1,750 (7.3%) | |
| Missing | 2,381 (0.1%) | 242 (0.2%) | 160 (0.2%) | 64 (0.1%) | 67 (0.3%) | |
| **Apgar score at 5-minute** | |  |  |  | |  |
| ≥7 | 2,188,621 (98.1%) | 139,646 (97.1%) | 103,944 (97.7%) | 47,028 (97.2%) | 22,540 (94.2%) | |
| <7 | 22,285 (1.0%) | 2,397 (1.7%) | 1,253 (1.2%) | 799 (1.7%) | 997 (4.2%) | |
| Missing | 21,204 (0.9%) | 1,703 (1.2%) | 1,184 (1.1%) | 536 (1.1%) | 396 (1.7%) | |
| **Neonatal asphyxia-related comorbidities** | 93,892 (4.2%) | 9,755 (6.8%) | 5,967 (5.6%) | 3,284 (6.8%) | 3,191 (13.3%) | |
| **Neonatal hypoglycemia** | 38,794 (1.7%) | 4,427 (3.1%) | 2,901 (2.7%) | 1,603 (3.3%) | 1,286 (5.4%) | |
| **Neonatal jaundice** | 99,213 (4.4%) | 8,892 (6.2%) | 6,078 (5.7%) | 3,076 (6.4%) | 2,118 (8.8%) | |
| **Abbreviations:** BMI-Body Mass Index; ADHD-attention deficit/ hyperactivity disorder. All results are presented as numbers and percentages (n, %). ^a^Available from 1992 onwards. ^b^Including gestational hypertension, pre-eclampsia (mild and severe), and eclampsia. | | | | | | |
