# Supplementary material for: Maternal intrahepatic cholestasis of pregnancy and neurodevelopmental conditions in offspring: A population-based cohort study of 2 million Swedish children
Source: PLoS Med. 2024 Jan 16;21(1):e1004331. doi: 10.1371/journal.pmed.1004331 (PMC10790993; doi:10.1371/journal.pmed.1004331)
Supplement: S3 Table — (DOCX) [file pmed.1004331.s010.docx]

**S3 Table.** The association between intrahepatic cholestasis of pregnancy and any neurodevelopmental conditions among those with identifiable full cousins and full siblings.

|  | **Full cohort^a^** | **Restricted to full cousins^b^** | **P (Difference)^c^** | **Full cousin analysis^d^** |
| --- | --- | --- | --- | --- |
| **Any diagnoses of ICP** |  |  |  |  |
| Total N | 2,375,856 | 791,759 |  | 138,467 |
| No. exposed cases | 713 | 231 |  | 220 |
| No. unexposed cases | 143,033 | 48,024 |  | 46,255 |
| OR (95% CI) | 1.22 (1.13-1.31) | 1.23 (1.07-1.41) | 0.89 | 1.24 (1.09-1.42) |
| **Diagnosed<28 weeks** |  |  |  |  |
| Total N | 2,365,822 | 788,437 |  | 137,897 |
| No. exposed cases | 43 | 16 |  | 15 |
| No. unexposed cases | 143,033 | 48,024 |  | 46,255 |
| OR (95% CI) | 2.38 (1.71-3.30) | 3.20 (1.82-5.63) | 0.19 | 2.99 (1.48-6.04) |
| **Diagnosed between 28-36 weeks** |  |  |  |  |
| Total N | 2 369 237 | 789,556 |  | 138,088 |
| No. exposed cases | 277 | 87 |  | 84 |
| No. unexposed cases | 143 033 | 48,024 |  | 46,255 |
| OR (95% CI) | 1.36 (1.20-1.54) | 1.36 (1.09-1.70) | 0.99 | 1.35 (1.08-1.67) |
| **Diagnosed ≥37 weeks** |  |  |  |  |
| Total N | 2 371 753 | 790,432 |  | 138 ,224 |
| No. exposed cases | 393 | 128 |  | 121 |
| No. unexposed cases | 143 033 | 48,024 |  | 46,255 |
| OR (95% CI) | 1.08 (0.97-1.20) | 1.07 (0.89-1.29) | 0.94 | 1.10 (0.93-1.31) |
|  |  |  |  |  |
|  | **Full cohort^a^** | **Restricted to full siblings^e^** | **P (Difference)^c^** | **Full sibling analysis^f^** |
| **Any diagnoses of ICP** |  |  |  |  |
| Total N | 2,375,856 | 1,711,498 |  | 185,323 |
| No. exposed cases | 713 | 470 |  | 379 |
| No. unexposed cases | 143,033 | 96,579 |  | 80,874 |
| OR (95% CI) | 1.22 (1.13-1.31) | 1.22 (1.11-1.34) | 0.85 | 1.04 (0.88-1.24) |
| **Diagnosed<28 weeks** |  |  |  |  |
| Total N | 2,365,822 | 1,704,451 |  | 184,500 |
| No. exposed cases | 43 | 28 |  | 21 |
| No. unexposed cases | 143,033 | 96,579 |  | 80 874 |
| OR (95% CI) | 2.38 (1.71-3.30) | 2.61 (1.73-3.93) | 0.43 | 1.92 (0.92-4.02) |
| **Diagnosed between 28-36 weeks** |  |  |  |  |
| Total N | 2,369,237 | 1,706,705 |  | 184,791 |
| No. exposed cases | 277 | 186 |  | 147 |
| No. unexposed cases | 143,033 | 96,579 |  | 80,874 |
| OR (95% CI) | 1.36 (1.20-1.54) | 1.49 (1.28-1.73) | 0.05 | 1.07 (0.82-1.39) |
| **Diagnosed ≥37 weeks** |  |  |  |  |
| Total N | 2,371,753 | 1,708,780 |  | 184,968 |
| No. exposed cases | 393 | 256 |  | 211 |
| No. unexposed cases | 143 033 | 96,579 |  | 80,874 |
| OR (95% CI) | 1.08 (0.97-1.20) | 1.03 (0.91-1.17) | 0.22 | 0.99 (0.81-1.22) |

**Abbreviations:** ICP-Intrahepatic cholestasis of pregnancy; NDC-Neurodevelopmental conditions; ADHD-Attention deficit/hyperactivity disorder.

^a^ Logistic regression was used for analysis, with standard errors computed using the robust (sandwich) method. Models were adjusted for child’s sex, birthyear, maternal age, highest parental education level, maternal birth country, birth order, maternal psychiatric history, and birth month.

^b^ Restricted to individuals with identifiable maternal cousins. Repeating the analyses in “a”.

^c^ Adjusted Wald test. A comparison of estimates in the index analysis and in “a”.

^d^ Conditional logistic regression. Adjust for all observed putative confounders which differed among relatives (i.e., child’s sex, birthyear, maternal age, highest parental education level, maternal birth country, birth order, maternal psychiatric history, and birth month).

^e^ Restricted to individuals with identifiable siblings. Repeating the analyses in “a”.

^f^ Conditional logistic regression. Adjust for all observed putative confounders which differed among relatives (e.g., not maternal education for full siblings).
