# Supplementary material for: Maternal intrahepatic cholestasis of pregnancy and neurodevelopmental conditions in offspring: A population-based cohort study of 2 million Swedish children
Source: PLoS Med. 2024 Jan 16;21(1):e1004331. doi: 10.1371/journal.pmed.1004331 (PMC10790993; doi:10.1371/journal.pmed.1004331)
Supplement: S4 Table — (DOCX) [file pmed.1004331.s011.docx]

**S4 Table.** The association between intrahepatic cholestasis of pregnancy and neurodevelopmental conditions after excluding those with intrahepatic cholestasis of pregnancy diagnosed at delivery and after excluding those born prematurely.

|  | **Main analysis**  (N=2,375,856)^a^ | **Exclude those diagnosed with ICP at birth** (N=2,373,566; N_excluded_=2,290)^a^ | | **Exclude those with preterm birth** (N=2,257,768; N_excluded_= 118,088)^a^ | |
| --- | --- | --- | --- | --- | --- |
|  | **OR (95% CI)** | **OR (95% CI)** | **P (Difference)^b^** | **OR (95% CI)** | **P (Difference)^b^** |
| **Any diagnoses of ICP** |  |  |  |  |  |
| Any NDCs | 1.22 (1.13-1.31) | 1.26 (1.15-1.37) | 0.11 | 1.22 (1.12-1.33) | 0.91 |
| ADHD | 1.25 (1.14-1.36) | 1.28 (1.16-1.41) | 0.30 | 1.24 (1.13-1.36) | 0.76 |
| Autism | 1.20 (1.06-1.36) | 1.25 (1.09-1.44) | 0.22 | 1.21 (1.06-1.39) | 0.74 |
| Intellectual disability | 1.01 (0.82-1.24) | 1.04 (0.82-1.31) | 0.67 | 1.06 (0.84-1.33) | 0.32 |
| **Diagnosed<28 weeks** |  |  |  |  |  |
| Any NDCs | 2.38 (1.71-3.30) | 2.38 (1.71-3.30) | 0.82 | 2.60 (1.79-3.79) | 0.32 |
| ADHD | 2.46 (1.70-3.55) | 2.46 (1.70-3.55) | 0.59 | 2.73 (1.80-4.12) | 0.31 |
| Autism | 1.56 (0.82-2.94) | 1.56 (0.82-2.94) | 0.91 | 1.58 (0.74-3.36) | 0.94 |
| Intellectual disability | 2.34 (1.10-4.96) | 2.34 (1.10-4.96) | 0.69 | 1.99 (0.74-5.37) | 0.63 |
| **Diagnosed between 28-36 weeks** |  |  |  |  |  |
| Any NDCs | 1.36 (1.20-1.54) | 1.34 (1.18-1.53) | 0.45 | 1.41 (1.20-1.65) | 0.48 |
| ADHD | 1.36 (1.18-1.57) | 1.33 (1.14-1.54) | 0.25 | 1.37 (1.14-1.64) | 0.90 |
| Autism | 1.32 (1.07-1.62) | 1.30 (1.05-1.60) | 0.59 | 1.37 (1.06-1.77) | 0.62 |
| Intellectual disability | 0.97 (0.68-1.39) | 0.93 (0.64-1.36) | 0.49 | 1.01 (0.63-1.60) | 0.83 |
| **Diagnosed ≥37 weeks** |  |  |  |  |  |
| Any NDCs | 1.08 (0.97-1.20) | 1.10 (0.97-1.25) | 0.55 | 1.10 (1.00-1.23) | <0.001 |
| ADHD | 1.12 (1.00-1.26) | 1.15 (0.99-1.33) | 0.62 | 1.14 (1.01-1.28) | <0.001 |
| Autism | 1.12 (0.95-1.32) | 1.19 (0.98-1.45) | 0.27 | 1.14 (0.97-1.35) | <0.001 |
| Intellectual disability | 0.96 (0.73-1.26) | 1.01 (0.73-1.41) | 0.59 | 1.04 (0.80-1.37) | <0.001 |

**Abbreviations:** ICP-Intrahepatic cholestasis of pregnancy; NDC-Neurodevelopmental disorder; ADHD-Attention deficit/hyperactivity disorder.

^a^ Logistic regression models with standard errors computed using the robust (sandwich) method. Adjusted for child’s sex, birthyear, maternal age, highest parental education level, maternal birth country, birth order, maternal psychiatric history, and birth month.

^b^ Adjusted Wald test. A comparison of estimates in the index analysis and the estimates in the main analysis.
