# Supplementary material for: Maternal intrahepatic cholestasis of pregnancy and neurodevelopmental conditions in offspring: A population-based cohort study of 2 million Swedish children
Source: PLoS Med. 2024 Jan 16;21(1):e1004331. doi: 10.1371/journal.pmed.1004331 (PMC10790993; doi:10.1371/journal.pmed.1004331)
Supplement: S5 Table — The association between intrahepatic cholestasis of pregnancy and neurodevelopmental conditions while adjusting for maternal BMI. (DOCX) [file pmed.1004331.s012.docx]

**S5 Table.** The association between intrahepatic cholestasis of pregnancy and neurodevelopmental conditions after excluding those with intrahepatic cholestasis of pregnancy diagnosed at delivery, while adjusting for maternal BMI.

|  | **Main analysis**  (N=2,375,856)^a^ | **Exclude those born < 1992** (N=1,822,626; N_excluded_ = 553,230)^b^ | **Adjusted for maternal BMI using the missing-as-indicator approach among those born ≥1992** (N=1,822,626; N_excluded_ = 553,230)^c^ | **Adjusted for maternal BMI using the complete case analysis among those born ≥1992** (N=1,565,501; N_excluded_ = 810,355)^d^ | **Adjusted for maternal BMI using the inverse probability weights among those born ≥1992** (N=1,822,626; N_excluded_ = 553,230)**^e^** | **Adjusted for maternal BMI using the multiple imputation approach among those born ≥1992** (N=1,822,626; N_excluded_ = 553,230)^f^ |
| --- | --- | --- | --- | --- | --- | --- |
|  | **OR (95% CI)** | **OR (95% CI)** | **OR (95% CI)** | **OR (95% CI)** | **OR (95% CI)** | **OR (95% CI)** |
| **Any diagnoses of ICP** |  |  |  |  |  |  |
| Any NDCs | 1.22 (1.13-1.31) | 1.18 (1.09-1.28) | 1.18 (1.09-1.28) | 1.18 (1.08-1.29) | 1.20 (1.06-1.36) | 1.18 (1.09-1.28) |
| ADHD | 1.25 (1.14-1.36) | 1.20 (1.10-1.32) | 1.20 (1.09-1.32) | 1.20 (1.08-1.32) | 1.23 (1.07-1.42) | 1.20 (1.10-1.32) |
| Autism | 1.20 (1.06-1.36) | 1.18 (1.04-1.35) | 1.18 (1.04-1.35) | 1.17 (1.01-1.36) | 1.22 (1.00-1.48) | 1.18 (1.04-1.35) |
| Intellectual disability | 1.01 (0.82-1.24) | 0.97 (0.78-1.22) | 0.97 (0.77-1.21) | 0.93 (0.72-1.19) | 1.11 (0.80-1.55) | 0.97 (0.78-1.21) |
| **Diagnosed<28 weeks** |  |  |  |  |  |  |
| Any NDCs | 2.38 (1.71-3.30) | 2.23 (1.56-3.17) | 2.17 (1.53-3.09) | 2.44 (1.67-3.56) | 1.96 (1.08-3.56) | 2.19 (1.54-3.11) |
| ADHD | 2.46 (1.70-3.55) | 2.26 (1.52-3.35) | 2.20 (1.48-3.27) | 2.44 (1.59-3.73) | 2.11 (1.05-4.22) | 2.22 (1.49-3.29) |
| Autism | 1.56 (0.82-2.94) | 1.67 (0.88-3.16) | 1.63 (0.86-3.09) | 1.91 (0.97-3.76) | 1.08 (0.46-2.51) | 1.64 (0.86-3.11) |
| Intellectual disability | 2.34 (1.10-4.96) | 1.85 (0.76-4.52) | 1.81 (0.74-4.41) | 1.88 (0.69-5.08) | 1.84 (0.51-6.66) | 1.82 (0.75-4.44) |
| **Diagnosed between 28-36 weeks** |  |  |  |  |  |  |
| Any NDCs | 1.36 (1.20-1.54) | 1.30 (1.14-1.49) | 1.29 (1.13-1.47) | 1.23 (1.05-1.42) | 1.39 (1.15-1.68) | 1.29 (1.13-1.48) |
| ADHD | 1.36 (1.18-1.57) | 1.29 (1.11-1.51) | 1.28 (1.10-1.49) | 1.23 (1.03-1.46) | 1.35 (1.08-1.68) | 1.28 (1.10-1.50) |
| Autism | 1.32 (1.07-1.62) | 1.29 (1.04-1.60) | 1.27 (1.02-1.58) | 1.17 (0.91-1.49) | 1.47 (1.08-2.00) | 1.28 (1.03-1.58) |
| Intellectual disability | 0.97 (0.68-1.39) | 0.95 (0.65-1.39) | 0.93 (0.64-1.37) | 0.79 (0.51-1.25) | 1.26 (0.74-2.13) | 0.93 (0.64-1.37) |
| **Diagnosed ≥37 weeks** |  |  |  |  |  |  |
| Any NDCs | 1.08 (0.97-1.20) | 1.06 (0.95-1.19) | 1.07 (0.96-1.19) | 1.10 (0.97-1.23) | 1.04 (0.88-1.23) | 1.07 (0.96-1.19) |
| ADHD | 1.12 (1.00-1.26) | 1.10 (0.98-1.25) | 1.11 (0.98-1.25) | 1.12 (0.98-1.28) | 1.11 (0.92-1.34) | 1.11 (0.98-1.25) |
| Autism | 1.12 (0.95-1.32) | 1.10 (0.93-1.31) | 1.11 (0.93-1.32) | 1.14 (0.95-1.37) | 1.07 (0.83-1.39) | 1.11 (0.93-1.32) |
| Intellectual disability | 0.96 (0.73-1.26) | 0.94 (0.71-1.26) | 0.94 (0.71-1.26) | 0.96 (0.70-1.31) | 0.97 (0.62-1.53) | 0.94 (0.71-1.26) |

**Abbreviations:** ICP-Intrahepatic cholestasis of pregnancy; NDC-Neurodevelopmental disorder; ADHD-Attention deficit/hyperactivity disorder.

^a^ Logistic regression models with standard errors computed using the robust (sandwich) method. Adjusted for child’s sex, birthyear, maternal age, highest parental education level, maternal birth country, birth order, maternal psychiatric history, and birth month.

^b^ Maternal BMI at the first antenatal visit has been measured since 1992. Before 1992, maternal BMI was calculated by subtracting total gestational weight gain from the last weight measurement before delivery. Therefore, we first repeated our main analyses among those born ≥1992. Adjusted for covariates in “a”.

^c^ Among those born ≥ 1992, 257,125 (14.11%) observations had missing data on maternal BMI. We replaced the missing values in BMI with a dummy category and replicated the analyses by adjusting for covariates in “a” and maternal BMI.

^d^ Complete case analysis. Among those born ≥ 1992, 257,125 (14.11%) observations having missing data on maternal BMI were excluded. Adjusted for covariates in “a” and maternal BMI.

^e^ Among those born ≥ 1992, 257,125 (14.11%) observations had missing data on maternal BMI. We replaced the missing values in BMI with a dummy category and replicated the analyses by adjusting for covariates in “a” and maternal BMI. Analyses were adjusted for potential bias from missing maternal BMI data using Inverse Probability Weighting (IPW). The weights were calculated based on the propensity of having observed maternal BMI values, modeled using child’s sex, birthyear, maternal age, highest parental education level, maternal birth country, birth order, maternal psychiatric history, and birth month. This approach reweights the observed data to be more representative of the entire sample, aiming to mitigate bias introduced by the missing BMI values.

^f^ Multiple imputation for maternal BMI, where 20 imputed datasets were generated to address missing data in maternal BMI (categorical variable) among those born ≥ 1992. Imputations were performed using multinomial imputation. The imputation model included any NDCs, maternal ICP, child’s sex, birthyear, maternal age, highest parental education level, maternal birth country, birth order, maternal psychiatric history, and birth month. Analyses were conducted separately for each imputed dataset and results were combined using Rubin's rules.
