# Supplementary material for: Maternal intrahepatic cholestasis of pregnancy and neurodevelopmental conditions in offspring: A population-based cohort study of 2 million Swedish children
Source: PLoS Med. 2024 Jan 16;21(1):e1004331. doi: 10.1371/journal.pmed.1004331 (PMC10790993; doi:10.1371/journal.pmed.1004331)
Supplement: Attachment — Submitted filename: Author response.docx [file pmed.1004331.s013.docx]

**REQUESTS FROM THE EDITORS:**

**Thank you for your patience, and apologies for the delay in providing you with an editorial decision. As you will see from the reviewer comments, adjustment is required for gestational age in your analyses. Please respond to the reviewer and editorial comments in full before resubmitting your manuscript, which will be re-reviewed.**

**Authors’ response:** We are immensely grateful for these thoughtful comments, both from the editor and reviewers. With the invaluable feedback received and the subsequent revisions carried out in accordance with the suggestions, we are confident that our manuscript has significantly improved.

**Comment from the Academic Editor:**

1) **The authors need to fully adjust for GA; please see e.g. PMID 20543995 which addresses the continuous relationship between GA and special educational needs.**

**Authors’ response:** We greatly appreciate the opportunity to elaborate on this crucial point.

It may seem intuitive to adjust for gestational age since individuals with ICP typically experience an (iatrogenic) early delivery. However, gestational age must occur subsequent to any ICP debut – and therefore cannot confound the estimated association between ICP and offspring health. Gestational age could serve as a mediator or collider (i.e., an intermediate state on the path between ICP and offspring neurodevelopment). In other words, it might be possible that if there is truly a causal effect of ICP on offspring neurodevelopment, this effect occurs through iatrogenic early delivery, a known risk factor for adverse neurodevelopment, as noted by the editor. Indeed, when approaching this work, this was one of our working theories for why ICP-exposed children would be at a greater risk of developing neurodevelopmental conditions, which is why we outlined it in the original submission (Page 3, Line 75-76).

Therefore, adjusting for gestational age is not an appropriate analytical strategy; it will underestimate the total causal effect if there is mediation, and it may induce spurious correlations if gestational age is considered a collider. We note that this critical consideration is not unique to ICP. We have encountered similar questions when examining the effects of cesarean section on offspring obesity (published in PLOS Medicine: <https://doi.org/10.1371/journal.pmed.1002996> ). In that study we recognized that gestational age might indeed act as a collider and omitted its adjustment in supplemental analyses.

Nevertheless, as several reviewers and the editor expressed concerns about this issue, we have provided a description of the phenomenon, along with a directed acyclic graph (now S5 Figure) illustrating how these factors may be involved in neurodevelopment (page 11; line 254-259):

*“In our main analysis, we did not adjust for gestational age, as we consider it to be a mediator or a collider (S5 Figure). However, in complementary analyses not registered in the protocol, we adjusted for gestational age using three different approaches: as a linear term in weeks, as a categorical term (<37, 37-, 38-, 39-, 40-, 41-, 42-), and by employing restricted cubic splines with 4 knots placed at the 5th, 35th, 65th, and 95th percentile (S6 Table).”*

We have repeated our analysis adjusting for gestational age, finding that this adjustment does not seem to explain our findings; the findings are slightly attenuated, as one would expect if adjustment were made for a mediator, but the qualitative conclusion remains the same. However, given the complexities described above and the strong limitations of adjusting for downstream factors, we will retain this analysis as supplementary for readers interested in such an analysis. We briefly comment on these findings in the result section (page 15: line 354-356):

*“Adjusting for gestational age slightly attenuated the observed associations but did not alter the qualitative conclusion; early ICP was associated with neurodevelopmental conditions irrespective of controlling for gestational age (S6 Table), albeit with slightly less precision.”*

For convenience, see excerpt from new S6 Table:

|  |  | **Adjusting for gestational age as:** | | |
| --- | --- | --- | --- | --- |
|  | **Main analysis**  **(As reported in main article)** | **Linear term** | **Categorical term** | **Restricted cubic splines** |
|  | **OR (95% CI)** | **OR (95% CI)^b^** | **OR (95% CI)^c^** | **OR (95% CI)^d^** |
| **ICP diagnosed<28 weeks** |  |  |  |  |
| Any NDCs | 2.38 (1.71-3.30) | 2.13 (1.53-2.97) | 2.14 (1.54-2.98) | 2.09 (1.50-2.91) |
| ADHD | 2.46 (1.70-3.55) | 2.27 (1.57-3.28) | 2.28 (1.58-3.30) | 2.24 (1.55-3.24) |
| Autism | 1.56 (0.82-2.94) | 1.39 (0.73-2.64) | 1.40 (0.74-2.66) | 1.36 (0.71-2.58) |
| Intellectual disability | 2.34 (1.10-4.96) | 1.73 (0.81-3.70) | 1.75 (0.82-3.71) | 1.68 (0.78-3.60) |
| **Abbreviations:** ICP-Intrahepatic cholestasis of pregnancy; NDC-Neurodevelopmental disorder; ADHD-Attention deficit/hyperactivity disorder.  ^a^Adjusted for the same covariates as the main model, and gestational age in weeks as a linear term.  ^b^Adjusted for the same covariates as the main model, and gestational age in weeks as a linear term.  ^c^Adjusted for the same covariates as the main model, and gestational age in weeks as a categorical term (<37, 37-, 38-, 39-, 40-, 41-, 42-).  ^d^Adjusted for the same covariates as the main model, and gestational age in weeks using restricted cubic splines with 4 knots placed at the 5^th^, 35^th^, 65^th^, and 95^th^ percentile. | | | | |

2) **Throughout the paper, please adapt reference call-outs to the following style: "... every year [1,2]." (noting the absence of spaces within the square brackets).**

**Authors’ response:** Amended as requested.

3) **Please define all abbreviations at first use e.g. CI, IQR, etc.**

**Authors’ response:** Amended as requested.

4) **Abstract: Please structure your abstract using the PLOS Medicine headings (Background, Methods and Findings (combined in one subsection), Conclusions). Please ensure that the Abstract is written as prose.**

**Authors’ response:** Amended as requested.

5) **Abstract Background: The final sentence should clearly state the study question.**

**Authors’ response:** Amended as requested.

6) **Abstract Methods and Findings: Please provide brief demographic details of the study population (e.g. sex, ethnicity, etc)**

**Authors’ response:** Amended as requested.

7) **Please include the important dependent variables that are adjusted for in the analyses.**

**Authors’ response:** Amended as requested.

8) **In the last sentence of the Abstract Methods and Findings section, please describe 2-3 of the main limitations of the study's methodology.**

**Authors’ response:** Amended as requested. The main limitations include the observational nature of our study, our inability to study recent ICP therapeutics, and the lack of bile acid measures.

9) **Please define CI at first use.**

**Authors’ response:** Amended as requested.

10) **Please define “term” in terms of gestational age.**

**Authors’ response:** Amended as requested.

11) **Please clarify what is being compared for the ORs presented for maternal cousins and full siblings.**

**Authors’ response:** Amended as requested.

12) **Abstract Conclusions: Please begin your Abstract Conclusions with "In this study, we observed ..." or similar, to summarize the main findings from your study, without overstating your conclusions. Please emphasize what is new and address the implications of your study, being careful to avoid assertions of primacy.**

**Authors’ response:** Amended as requested.

13) **Author Summary: At this stage, we ask that you include a short, non-technical Author Summary of your research to make findings accessible to a wide audience that includes both scientists and non-scientists. The Author Summary should immediately follow the Abstract in your revised manuscript. This text is subject to editorial change and should be distinct from the scientific abstract. Please see our author guidelines for more information:** [**https://eur01.safelinks.protection.outlook.com/?url=https%3A%2F%2Fjournals.plos.org%2Fplosmedicine%2Fs%2Frevising-your-manuscript%23loc-author-summary&data=05%7C01%7Cviktor.ahlqvist%40ki.se%7C684845aa589c4cb3c14a08dbc0156bb8%7Cbff7eef1cf4b4f32be3da1dda043c05d%7C0%7C0%7C638314970463489917%7CUnknown%7CTWFpbGZsb3d8eyJWIjoiMC4wLjAwMDAiLCJQIjoiV2luMzIiLCJBTiI6Ik1haWwiLCJXVCI6Mn0%3D%7C3000%7C%7C%7C&sdata=wwlMH5d1FIOztYuC5YkwLh3%2FaSbheL%2FE%2Bzv3DFwe9vk%3D&reserved=0**](https://eur01.safelinks.protection.outlook.com/?url=https%3A%2F%2Fjournals.plos.org%2Fplosmedicine%2Fs%2Frevising-your-manuscript%23loc-author-summary&data=05%7C01%7Cviktor.ahlqvist%40ki.se%7C684845aa589c4cb3c14a08dbc0156bb8%7Cbff7eef1cf4b4f32be3da1dda043c05d%7C0%7C0%7C638314970463489917%7CUnknown%7CTWFpbGZsb3d8eyJWIjoiMC4wLjAwMDAiLCJQIjoiV2luMzIiLCJBTiI6Ik1haWwiLCJXVCI6Mn0%3D%7C3000%7C%7C%7C&sdata=wwlMH5d1FIOztYuC5YkwLh3%2FaSbheL%2FE%2Bzv3DFwe9vk%3D&reserved=0) **In the final bullet point of ‘What Do These Findings Mean?’, please describe the main limitations of the study in non-technical language.**

**Authors’ response:** Amended as requested.

14) **Introduction: Line 77 - please temper assertions of primacy by adding ‘to the best of our knowledge’ or similar**

**Authors’ response:** Amended as requested.

15) **Methods: Did your study have a prospective protocol or analysis plan? Please state this (either way) early in the Methods section. If a prospective analysis plan (from your funding proposal, IRB or other ethics committee submission, study protocol, or other planning document written before analyzing the data) was used in designing the study, please include the relevant prospectively written document with your revised manuscript as a Supporting Information file to be published alongside your study, and cite it in the Methods section. A legend for this file should be included at the end of your manuscript. If no such document exists, please make sure that the Methods section transparently describes when analyses were planned, and if/when reported analyses differed from those that were planned. Changes in the analysis-- including those made in response to peer review comments-- should be identified as such in the Methods section of the paper, with rationale. If a reported analysis was performed based on an interesting but unanticipated pattern in the data, please be clear that the analysis was data-driven.**

**Authors’ response:** The study included a prospective protocol and analysis plan that underwent review by the data approval board for Psychiatry Sweden (new S2 Appendix). In the proposal, we outlined a brief background, research questions, main analysis, and methods. All sensitivity analyses were either standard approaches typically employed by our research teams in registry studies or were developed during the research process. This protocol and analysis plan is now described in detail in the manuscript (page 9; line 200-203):

*“The main analysis was prospectively planned (S2 Appendix), and all sensitivity analyses were developed during the research process.”*

Furthermore, we also clarify that the inclusion of the crude analysis, adjustments for gestational age, and the imputation of missing data was suggested by the reviewers (page 9; line 200-203):

*“Reviewers requested a crude analysis, an analysis that adjusts for gestational age and an analysis with detailed imputation of maternal pre-pregnancy BMI; these analyses were therefore not part of our original prospective plan.”*

16) **Please add the following statement, or similar, to the Methods: "This study is reported as per the Strengthening the Reporting of Observational Studies in Epidemiology (STROBE) guideline (S1 Checklist)." The STROBE guideline can be found here:** [**https://eur01.safelinks.protection.outlook.com/?url=http%3A%2F%2Fwww.equator-network.org%2Freporting-guidelines%2Fstrobe%2F&data=05%7C01%7Cviktor.ahlqvist%40ki.se%7C684845aa589c4cb3c14a08dbc0156bb8%7Cbff7eef1cf4b4f32be3da1dda043c05d%7C0%7C0%7C638314970463489917%7CUnknown%7CTWFpbGZsb3d8eyJWIjoiMC4wLjAwMDAiLCJQIjoiV2luMzIiLCJBTiI6Ik1haWwiLCJXVCI6Mn0%3D%7C3000%7C%7C%7C&sdata=H6Np06jla%2BkvyXD3mtVyHjozMDIAwWzaVz17nD2eC%2Bs%3D&reserved=0**](http://www.equator-network.org/reporting-guidelines/strobe/)

**Authors’ response:** We previously included the STROBE-Checklist as supplementary material. We have now updated it in accordance with the comments from the editor and reviewers. Please refer to the new S1 Appendix.

17) **Results: Please present numerators and denominators for percentages in the Tables.**

**Authors’ response:** For Table 1, the Total N serves as the denominator, while the numbers in each cell represent the numerator, we have now clarified this in the footnote. For Table 2, we have added a separate row representing the denominator (i.e., *Total*). We have also rephrased the column labels to make it clearer that these are the numerators/denominators.

18) **For the adjusted analyses in Table 2, please also provide the unadjusted analyses.**

**Authors’ response:** We have now added a column to the table for the results of unadjusted analyses (crude models).

19) **Please indicate which factors are adjusted for, in the main text.**

**Authors’ response:** Amended as requested. Please see page 12; line 280-282.

20) **Line 209 - please revise to ‘strength of the association’**

**Authors’ response:** Amended as requested.

21) **Line 221 - the results presented in this paragraph do not correspond well with the results presented in Table 2, showing that there is only an association (after full adjustment) between ICP and autism between 28-36 weeks of pregnancy (and all diagnoses of ICP); and between ICP before 28 weeks of pregnancy and intellectual disability. Please revise this paragraph to better reflect the results presented in Table 2, especially the sentence “children born to mothers with ICP diagnosed before 28 weeks of gestation were more likely to be diagnosed with autism (adjusted OR 1.56, 95% CI 0.82-2.94)"**

**Authors’ response:** Thank you for highlighting this. We believe that our reporting of the results is an accurate representation of our findings – early ICP is associated with neurodevelopmental conditions in offspring. However, we realize that some of the neurodevelopmental *specific* findings are not always 'statistically significant' (even if, for the example the editor cites, early ICP is associated with a 56% higher odds). We have revised our manuscript throughout to clearly indicate whether our findings were statistically significant or not. Please refer to page 13-14; line 304-318.

22) **Please see** [**https://eur01.safelinks.protection.outlook.com/?url=https%3A%2F%2Fjournals.plos.org%2Fplosmedicine%2Fs%2Fsupporting-information&data=05%7C01%7Cviktor.ahlqvist%40ki.se%7C684845aa589c4cb3c14a08dbc0156bb8%7Cbff7eef1cf4b4f32be3da1dda043c05d%7C0%7C0%7C638314970463489917%7CUnknown%7CTWFpbGZsb3d8eyJWIjoiMC4wLjAwMDAiLCJQIjoiV2luMzIiLCJBTiI6Ik1haWwiLCJXVCI6Mn0%3D%7C3000%7C%7C%7C&sdata=FG%2BtTO1HBgCMPe1WEm7N4ZF11egkKAm35qa9wuFjaOo%3D&reserved=0**](https://journals.plos.org/plosmedicine/s/supporting-information) **for our supporting information guidelines; supplementary files should be referred to as Table S1, S2, Figure S1, S2, and so on.**

**Authors’ response:** Amended as requested. According to the link, it should be S1 Table, S1 Figure, and S1 Appendix.

23) **Line 230 - please provide the results for the analysis** **excluding those without siblings (N=515 550) in eTable4 Sensitivity analyses: Please revise this paragraph to reflect that associations were not similar for** **ICP before 28 weeks of pregnancy and intellectual disability when excluding preterm birth, children born before 1993 and after adjustment for maternal BMI.**

**Authors’ response:** We apologize for this oversight. The results referred to by the editor were from an early draft that was subsequently updated and included in S3 Table. In Table S3, we retained families with at least one full sibling pair and separately included individuals contributing to at least one cousin set. When replicating the standard analysis in both the sibling and cousin samples, we found no major differences, which provides some support for the generalizability of this analysis.

Upon further consideration, we have also revised S3 Table to exclude the outcome-discordant selection sample because it is not appropriate. These samples would partially control for shared factors and, therefore, do not provide informative analysis to judge the validity of family-based analysis.

Furthermore, to guide readers more easily, we have performed adjusted Wald tests to compare the results of the main analysis to those of the sensitivity analyses (reported in S3 and S4 Tables). Generally, there were not many statistically significant differences (P_Wald tests_> 0.05). Some differences were statistically significant, albeit the estimates did not differ much. This appeared to be true for the analysis adjusting for maternal BMI. However, this adjustment could not explain the observed association between early ICP and offspring NDC. This information has now been updated in the main text (page 14-15; line 322-341) and is also detailed in S3 and S4 Tables.

Finally, the editor has expressed a concern regarding an inconsistency in the results for early ICP and intellectual disability in some sensitivity analyses. We are not entirely sure what this refers to, as our findings indicate that the estimates remain consistent across various analyses. To address this concern comprehensively, we have now included adjusted Wald tests to evaluate the differences between these estimates. Importantly, we would like to emphasize that these differences are *not* statistically significant. While it's true that some associations are not statistically significant when considered in isolation, the primary purpose of this table is to facilitate comparisons across estimates.

Nonetheless, as per response to editorial comment 21, we can revise the text to highlight that individual estimates are not statistically significant if that is preferrable to the editor and PLOS Medicine. Please refer to page 14-15 line 322-341.

Below is an excerpt from table S3, which now incorporates adjusted Wald tests showing that results are not different:

|  | **Main analysis**  (N=2,375,856)^a^ | **Exclude those diagnosed with ICP at birth** (N=2,373,566; N_excluded_=2,290)^a^ | | **Exclude those with preterm birth** (N=2,257,768; N_excluded_= 118,088)^a^ | |
| --- | --- | --- | --- | --- | --- |
| **Diagnosed<28 weeks** | **OR (95% CI)** | **OR (95% CI)** | **P (Difference)^b^** | **OR (95% CI)** | **P (Difference)^b^** |
| Intellectual disability | 2.34 (1.10-4.96) | 2.34 (1.10-4.96) | 0.69 | 1.99 (0.74-5.37) | 0.63 |
| ^a^Logistic regression models with standard errors computed using the robust (sandwich) method. Adjusted for child’s sex, birthyear, maternal age, highest parental education level, maternal birth country, birth order, maternal psychiatric history, and birth month.  ^b^ Adjusted Wald test. A comparison of estimates in the index analysis and the estimates in the main analysis. | | | | | |

24) **Discussion: Please present and organize the Discussion as follows: a short, clear summary of the article's findings; what the study adds to existing research and where and why the results may differ from previous research; strengths and limitations of the study; implications and next steps for research, clinical practice, and/or public policy; one-paragraph conclusion.**

**Authors’ response:** Amended as requested. We have now revised the discussion section according to your comment.

25) **Please remove all subheadings within your Discussion e.g. Strengths and limitations.**

**Authors’ response:** Amended as requested.

26) **Figures: When a p value is given, please specify the statistical test used to determine it in the figure legend.**

**Authors’ response:** We have added to the footnote of Figure 2.

27) **Please define abbreviations used in the figure legend of each figure.**

**Authors’ response:** We have ensured that all abbreviations are available in figure the legends.

28) **Please indicate in the figure caption what is represented by the error bars.**

**Authors’ response:** We have added this to the caption of Figure 2.

29) **Tables: When a p value is given, please specify the statistical test used to determine it in the table legend.**

**Authors’ response:** We have added it to the legends of Table 2, and S3-S4 Table.

30) **Please define all abbreviations used in the table legend of each table.**

**Authors’ response:** We have ensured that all abbreviations are available in table the legends.

31) **Please provide the unadjusted comparisons as well as the adjusted comparisons in Table 2.**

**Authors’ response:** We have now added another column in Table 2 for unadjusted results (crude models).

32) **References: Please ensure that journal name abbreviations match those found in the National Center for Biotechnology Information (NCBI) databases (**[**https://eur01.safelinks.protection.outlook.com/?url=http%3A%2F%2Fwww.ncbi.nlm.nih.gov%2Fnlmcatalog%2Fjournals&data=05%7C01%7Cviktor.ahlqvist%40ki.se%7C684845aa589c4cb3c14a08dbc0156bb8%7Cbff7eef1cf4b4f32be3da1dda043c05d%7C0%7C0%7C638314970463489917%7CUnknown%7CTWFpbGZsb3d8eyJWIjoiMC4wLjAwMDAiLCJQIjoiV2luMzIiLCJBTiI6Ik1haWwiLCJXVCI6Mn0%3D%7C3000%7C%7C%7C&sdata=MyHLW%2F8CkbKJ7acGez%2BI0ZKdqE0q5JGRDjybG7d6b58%3D&reserved=0**](http://www.ncbi.nlm.nih.gov/nlmcatalog/journals)**), and are appropriately formatted and capitalised. Six authors should be listed prior to ‘et al’.**

**Authors’ response:** Amended as requested.

33) **Please also see** [**https://eur01.safelinks.protection.outlook.com/?url=https%3A%2F%2Fjournals.plos.org%2Fplosmedicine%2Fs%2Fsubmission-guidelines%23loc-references&data=05%7C01%7Cviktor.ahlqvist%40ki.se%7C684845aa589c4cb3c14a08dbc0156bb8%7Cbff7eef1cf4b4f32be3da1dda043c05d%7C0%7C0%7C638314970463489917%7CUnknown%7CTWFpbGZsb3d8eyJWIjoiMC4wLjAwMDAiLCJQIjoiV2luMzIiLCJBTiI6Ik1haWwiLCJXVCI6Mn0%3D%7C3000%7C%7C%7C&sdata=Fa%2BdmyW0Y0FxR7mjEdsUzdscAupHEh4sra5jzcMUF%2Fc%3D&reserved=0**](https://journals.plos.org/plosmedicine/s/submission-guidelines#loc-references) **for further details on reference formatting.**

**Authors’ response:** Amended as requested.

**REVIEWER COMMENTS:**

**Reviewer #1:**

1) **Thank you for inviting me to review this manuscript, which uses a retrospective national dataset and linked coded diagnostic terms to interrogate any associations between ICP and offspring neurodevelopmental disorders (NDD). It is a useful and important topic, which attempts to answer a question that has not previously been adequately addressed within the literature, and also addresses questions that I have heard asked by affected families within PPI engagement. This study makes use of the excellent Swedish data collection at a population level, and is an appropriate population and study design to attempt to address this question.**

**Authors’ response:** We are grateful for your thoughtful review, and we appreciate the recognition of the importance of our work.

2) **It is important to recognise that, once this study is published, its simplest message will be interpreted across the patient community as being that "ICP causes NDD", which still cannot be proven by this retrospective approach demonstrating association rather than causation, and thus there is a critical importance that all co-variates are accounted for appropriately, and a very clear message is presented through from the abstract about this limitation.**

**Authors’ response:** We concur with this point. In fact, we believe it’s crucial to emphasize that our study is observational and cannot establish causality. Strong claims of causality require further replication, preferably using different types of data, more comprehensive phenotypes (such as measured bile acids), and various study designs. We hope that our study will inspire others to conduct further research in this important area.

Furthermore, we agree that a simple interpretation of our observational findings might lead laypersons to conclude that “ICP causes NDD”. To prevent such a misinterpretation, we reviewed our study and ensured that we consistently avoid making such strong claims.

Finally, while we have made extensive efforts to address confounding, both through measured covariates and by employing maternal cousin comparisons and full sibling comparisons, we acknowledge the inherent limitations of observational studies. Therefore, and in alignment with PLOS Medicine reporting preferences, we have now included this limitation in the abstract (page 3; line 60-62):

*“The primary limitations of this study include its observational design, the absence of data on ICP therapeutics, and the lack of bile acid measures.”*

3) **The authors have taken multiple approaches to account for the impact of co-variates on their overall findings. However, the major concern that I have with their findings is that they have not appropriately accounted for gestational age at birth - excluding those babies born preterm simply does not exclude the impact of advancing gestational age at birth being inversely associated with NDD even after 37/40. There is a plethora of studies demonstrating this, and it is one of the main counselling factors underpinning recommendations regarding planned gestational age at birth; Swedish population studies have also beautifully demonstrated the shorter term impacts of early term birth on many neonatal outcomes that plausibly could affect later NDD (Mitha et al. J Pediatrics 2021; 223: 43-50.e5). I therefore would recommend that the authors include gestational age at birth as a continuous variable in their statistical models to confirm that this does not impact their conclusion before considering accepting the manuscript.**

**Authors’ response:** Thank you for highlighting this important point, which has also been raised by other reviewers and editors (see also our response 1 to the editor). Our team has been working on this topic for a long time, and we have published several works on the links between gestational age/prematurity and neurodevelopmental outcomes (see for example, PMID: 27818373, 29214412 & 28898924). We concur that prematurity is a well-established risk factor for adverse fetal and neurodevelopmental outcomes.

In fact, our interest in investigating ICP’s potential impact on offspring neurodevelopment was partly driven by our observations of the rate of iatrogenic prematurity among affected pregnancies. We hypothesized that prematurity might serve as a mediator, connecting ICP and adverse offspring neurodevelopment (in addition to other complications of ICP). It's important to note that gestational age and prematurity cannot confound the relationship between ICP and offspring neurodevelopment, as gestational age must occur after the onset of ICP.

Given that gestational age can only act as a mediator or a collider, it is not appropriate to adjust for it in our primary analysis. Doing so would attenuate (bias towards the null) any true effect of ICP on neurodevelopmental disorders if gestational age indeed operates as a mediator or could lead to spurious correlations if gestational age functions as a collider. To illustrate this, consider the directed acyclic graph below (now included in online supplement- S5 Figure), where A) we don’t adjust for gestational age, and the effect of ICP on NDC is unbiased, B) we adjust for gestational age and block an effect and bias the estimate toward the null, and C) there is a residual confounder of gestational age and NDCs, and gestational age act as a collider, leading to biased effect estimates with unpredictable directions:

However, we acknowledge that not everyone may approach inference from this formal perspective (causal inference), where concepts such as mediators and colliders are well known. To address this, we have conducted additional analyses where we: 1) adjusted for gestational age in weeks as a linear term, 2) adjusted for categories of gestational weeks, and 3) adjusted for gestational age using restricted cubic splines to model it in a non-linear fashion. The results of these analyses are now presented in S6 Table. We briefly comment on these findings in the result section (page 15; line 354-356):

*“Adjusting for gestational age slightly attenuated the observed associations but did not alter the qualitative conclusion; early ICP was associated with neurodevelopmental conditions irrespective of controlling for gestational age (S6 Table), albeit with slightly less precision.”*

For reviewer convenience, we provide an excerpt of the early ICP findings below:

|  |  | **Adjusting for gestational age as:** | | |
| --- | --- | --- | --- | --- |
|  | **Main analysis**  **(As reported in main article)** | **Linear term** | **Categorical term** | **Restricted cubic splines** |
|  | **OR (95% CI)** | **OR (95% CI)^b^** | **OR (95% CI)^c^** | **OR (95% CI)^d^** |
| **ICP diagnosed<28 weeks** |  |  |  |  |
| Any NDCs | 2.38 (1.71-3.30) | 2.13 (1.53-2.97) | 2.14 (1.54-2.98) | 2.09 (1.50-2.91) |
| ADHD | 2.46 (1.70-3.55) | 2.27 (1.57-3.28) | 2.28 (1.58-3.30) | 2.24 (1.55-3.24) |
| Autism | 1.56 (0.82-2.94) | 1.39 (0.73-2.64) | 1.40 (0.74-2.66) | 1.36 (0.71-2.58) |
| Intellectual disability | 2.34 (1.10-4.96) | 1.73 (0.81-3.70) | 1.75 (0.82-3.71) | 1.68 (0.78-3.60) |
| **Abbreviations:** ICP-Intrahepatic cholestasis of pregnancy; NDC-Neurodevelopmental disorder; ADHD-Attention deficit/hyperactivity disorder.  ^a^Adjusted for the same covariates as the main model, and gestational age in weeks as a linear term.  ^b^Adjusted for the same covariates as the main model, and gestational age in weeks as a linear term.  ^c^Adjusted for the same covariates as the main model, and gestational age in weeks as a categorical term (<37, 37-, 38-, 39-, 40-, 41-, 42-).  ^d^Adjusted for the same covariates as the main model, and gestational age in weeks using restricted cubic splines with 4 knots placed at the 5^th^, 35^th^, 65^th^, and 95^th^ percentile. | | | | |

To provide further context on this analysis, we have included explanations and refer readers to the directed acyclic graph so they can better understand our rationale for not adjusting for gestational age in our primary analysis:

*“In our main analysis, we did not adjust for gestational age, as we consider it to be a mediator or a collider (S5 Figure). However, in complementary analyses not registered in the protocol, we adjusted for gestational age using three different approaches: as a linear term in weeks, as a categorical term (<37, 37-, 38-, 39-, 40-, 41-, 42-), and by employing restricted cubic splines with 4 knots placed at the 5th, 35th, 65th, and 95th percentile (S6 Table).”*

4) **I have huge concerns that the risk of publishing this manuscript without absolute certainty of the findings will be that patients read the association between longer exposure to ICP and increased risk of NDD, and therefore request earlier birth to prevent this (which in actual fact might increase the risk of NDD and other consequences of preterm and early term iatrogenic birth).**

**Authors’ response:** We appreciate this important point, as we also address in the comments above (referring to causal statements, and gestational age). We concur that it is not appropriate that patients read our findings of duration of ICP exposure as “being delivered earlier may prevent this”. To further reinforce that this would not be appropriate, we have included the following section in the discussion (page 16-17; line 381-389):

*“*[Another potential mediator linking ICP to offspring neurodevelopment is preterm birth. However, we did not observe a higher proportion of preterm births among those diagnosed with early-onset ICP. Furthermore, excluding premature births only had a negligible effect on our estimates, suggesting that preterm birth is unlikely to explain the stronger associations observed with early-onset ICP.]

*</paragraph break>*

*It's important, however, to emphasize that preterm birth is a well-recognized risk factor for offspring neurodevelopmental conditions [reference to PMID: 27189265, 31949163 & 27110944]. Our findings regarding the length of exposure to ICP should not be misconstrued as an encouragement for earlier deliveries of these children. In fact, although we are not powered to formally examine mediation, it seems probable that the connection between early ICP and offspring neurodevelopmental conditions may involve shorter gestation; earlier (iatrogenic) delivery is not necessarily recommended because it may partly explain the observed excess risk, and we currently do not know if treatment (i.e., ursodeoxycholic acid) might mitigate the observed risks.* [Further investigations are warranted to better understand the underlying aetiology of early-onset ICP and the possible mechanisms that implicate it in offspring neurodevelopmental conditions.]*”*

5) **We know that patients with early onset ICP are more likely to give birth at earlier gestations, and so this finding is not unsurprising.**

**Authors’ response:** We appreciate your insightful comment. This indeed forms part of the justification for our study, as we mentioned in our previous responses. We also share the reviewer’s opinion of our findings. Indeed, it's somewhat surprising that this relationship hasn't been studied before (because it is logically deducible).

It's true that individuals exposed to ICP often experience earlier deliveries, and both premature and early-term births can increase the risk of neurodevelopmental conditions in offspring. Therefore, it's reasonable to consider a shorter gestational duration as a potential mediator in the pathway connecting ICP to offspring NDCs. For a more comprehensive discussion of adjusting for gestational age, please see our responses to Comment 3 above and Editor's Comment 1.

6) **Similarly, it is very easy to look through the (well presented) Table 1 and Supplementary Table and come to vastly different conclusions if all of these factors are not accounted for in the final analyses: I would suggest from these data that: 1) More recent birth year 2) Caesarean birth 3) Induction of labour 4) Overweight / obesity or missing BMI data etc may be more associated with ICP and / or NDD - the authors have accounted for many of these factors in these models but it would be worth summarising in a table / figure which factors are independently associated with autism in separate models so that you are able to clearly follow why you have selected your various sensitivity analyses and confounders included. Otherwise, it may appear by chance that you have selected BMI as the factor to adjust by (supp table 4) rather than through logical selection of the most important confounding variable.**

**Authors’ response:** We are enthusiastic about the opportunity to provide further insights into our approach to selecting confounding variables. In line with contemporary practices in medicine, statistics, causal inference, and epidemiology, we meticulously choose covariates based on their *potential* to act as confounders. This involves considering whether a factor could theoretically have a causal influence on *both* the exposure and the outcome. Following established guidelines, like the STROBE guidelines (for instance, "Decisions about excluding or including variables should be guided by knowledge, or explicit assumptions, on causal relations. Inappropriate decisions may introduce bias, for example by including variables that are in the causal pathway between exposure and disease," as published in PLOS Medicine: <https://doi.org/10.1371/journal.pmed.0040297> ), we consciously avoid including mediators or colliders in our covariate selection. Such inclusion could introduce bias into our results.

Selection of covariates based on observed correlations, sometimes referred to as model-based covariate selection, is not compatible with causal inference. It might lead to the exclusion of important confounding variables, such as those that have a large impact on the exposure but a weak impact on the outcome. Conversely, it might include factors that aren't true confounders but happen to be correlated with both the exposure and the outcome, including potential mediators lying on the causal pathway. Nevertheless, we concur with the reviewer that some of the mentioned factors are correlated with the outcome, as is evident in S2 Table. However, this doesn't imply that they should be adjusted for in the analysis, as this necessitates thoughtful consideration of causal effects, as outlined above.

To enhance clarity in the covariates section, we have revised it to read:

"We collected a series of *potential confounding variables (factors theorized or confirmed to have a causal impact on both the exposure and the outcome)* from the Medical Birth Register..."

Finally, as per the request of reviewer 2, we have now included references for each putative confounder.

7) **In summary, I think that this is an important and useful manuscript, but would not recommend publication in its current format without appropriate correction as described above, most importantly treating gestation of birth as a continuous variable.**

**Authors’ response:** We sincerely appreciate your insightful comments on our manuscript. Your feedback has been instrumental in strengthening the quality of our work, and we would like to assure you that we have taken your concerns about gestational age into careful consideration.

As we have outlined in our responses, we have provided a thorough explanation of how we approach gestational age and why we believe it is inappropriate to adjust for it in our primary analysis.

However, we acknowledge your concern, and we recognize that others may share an interest in a gestational age-adjusted analysis. In response to this, we have included additional analyses in our revised manuscript that adjust for gestational age, as you suggested. The findings of this analysis revealed that adjusting for gestational age slightly attenuated the associations between maternal ICP and offspring NDCs, but the qualitative conclusion did not change; early ICP is associated with offspring neurodevelopmental conditions.

We hope that these modifications address your concerns and provide a more comprehensive description of our research. Your input has been invaluable in improving the clarity and rigor of our study.

8) **Once published, the manuscript risks reactionary concerns from those patients and clinicians who will read only the abstract and headline conclusion, and risk detrimental care decisions for the pregnancy which may be difficult to reverse given the relatively limited datasets with this duration of follow up that currently could be used to address this question.**

**Authors’ response:** We appreciate the concern raised by the clinical reviewer and would like to emphasize our commitment to responsible and clear communication in our publication.

In our Abstract, Author's Summary, and Discussion of limitations, we have made it a point to explicitly acknowledge the study's constraints, especially regarding the interpretation of causality. This serves as a preemptive measure to ensure that our findings are not misrepresented.

We have also conducted a series of sensitivity analyses. These analyses explore the impact of several biases, including gestational age at birth, offering a more comprehensive understanding of our results and their robustness.

Our presentation strategy is designed to prevent any unintended misinterpretation of our findings, especially among patients and clinical practitioners who might make care decisions based on the abstract and headline conclusion. We take the responsibility of clear communication seriously and are dedicated to ensuring the research's accuracy and reliability from both clinical and methodological perspectives.

We were particularly struck by the reviewer's concern about the potential implications of our findings, specifically the idea that they might lead women to request early delivery. We want to emphasize that we share this concern and have taken steps in our extended discussion to address it. It is our intention to ensure that this is not a misleading "headline conclusion" of our work.

**Reviewer #2:**

**Thanks for the opportunity to review your manuscript. My role is as a statistical reviewer, so my review concentrates on the study design, data, and analysis that are presented. I have put general questions first, followed by queries relevant to a specific section of the manuscript (with a page/paragraph reference).**

**This manuscript examines whether exposure to intrahepatic cholestasis during gestation is associated with later incidence of neurodevelopmental conditions (ADHD, autism, ID). The link between is ICP and ND is proposed based on links between ICP and other neonatal adverse events, and assuming ICP could similarly alter fetal brain development during a critical period. Data is taken from linked routinely collected healthcare data and registries from Sweden, and initially includes all children born between 1987 and 2010. Children not in the birth registry, or were not in country (and with data) for less than 5 years, non-singletons were excluded. Children with missing covariate information were also excluded (0.1 % of initial sample).  ICP (exposure) was identified from inpatient data, with date of diagnosis used for date of onset (and categorised into three categories). ND conditions (outcome) were ascertained from inpatient data, and dispensing for drugs used to treat ADHD. A combined ND outcome was used, as well as specific diagnoses as secondary outcomes. A range of covariates is considered, these are sourced from several different databases and includes in maternal, gestational, and birth information. Full details (i.e. ICD-10 codes) are provided in the appendix. The main analysis uses logistic regression, one minimally adjusted with sex and year of birth, and one that also considers more potential confounders. Several sensitivity analyses of the main analysis are considered, the rationale for these is explained well. In several secondary analyses, a conditional LR analysis of siblings and cousins who were discordant for ICP status were completed. There is a small association between ICP and ND in the main analysis, this shrinks back to no association in the full-sibling analysis. There is a 'dose-response' with period of ICP exposure, with more ND seen in those exposed to ICP from earlier in development. The association between ND and ICP seemed to be mostly driven by ADHD and Autism, with no robust association between ICP and ID. The results from the sensitivity analyses were consistent with the main analyses. Limitations of the study (e.g. limitations in the data collection) are clearly discussed. The figures presented in the manuscript are excellent - very clear and elegant.**

1) **P3, L75. I'm not sure what the right wording here is, but stillbirth can't be an intermediary between ICP and ND conditions. '**

**Authors’ response:** Thank you for bringing this to our attention. We have now rephrased the sentence to read (page 6; line 128):

“[However, ICP is associated with an elevated risk of adverse outcomes for infants, including stillbirth, preterm birth, and admission to neonatal care unit.] *Despite some of these factors, such as preterm birth,* [being associated with adverse neonatal outcomes, including neurodevelopmental conditions, to the best of our knowledge, there have been no previous studies examining neurodevelopmental conditions among offspring exposed to ICP during pregnancy.]”

2) **P4, L94. Why wasn't more recent data (2011-) used in the study?**

**Authors’ response**: We appreciate this point. Unfortunately, we cannot include more recent birth cohorts due to limitations imposed by the ethical permit granted by the Stockholm ethical committee for the "Psychiatry Sweden" project. This permit only covers the inclusion of birth cohorts up to 2010. As we mentioned in the original submission (page 13 Line 273-274), we encourage further replication of our study in more recent cohorts, especially where ICP treatments have become more widely used.

Finally, we note that a 'benefit' of studying older birth cohorts is that they have reached an age where they are diagnosed with the outcomes, which is a typical limitation faced by the analysis of contemporary birth cohorts.

3) **P4, L96. Were the children identified but on the birth registry likely to have been born outside of Sweden? Or are these non-matches from the data linkage process?**

**Authors’ response:** The Swedish Medical Birth Registry covers virtually all deliveries in Sweden, with a historically reported coverage of approximately 97-99% (see PMID: 36595114). The missing 2.9% of children in our specific study are likely missing for reasons that have been explored and described previously, namely that there have historically been lapses in reporting from specific hospitals (particularly in 1998, a birth cohort included in our study), home births are not recorded in the MBR (though these are a small proportion of births overall in Sweden), and there can be problems linking records identified by invalid personal identification numbers. The issue of invalid personal identification numbers most often arises for women who give birth in Sweden who are not yet registered as residents. For example, asylum seekers are given a temporary identification number that is often difficult to link to the personal number that is assigned when residency is granted, though this situation also arises for women who are EU-citizens (and thus are free to move to Sweden without a residence permit) and arrive in Sweden shortly before giving birth. We have not investigated the specific reasons for missingness of MBR records in our study, given that the proportion missing was well in line with the well-documented proportion missing that is to be expected.

We have now clarified in the text that the Swedish Medical Birth Register includes virtually all children born in Sweden and provided an updated reference for this (page 7; line 151-152):

“We excluded children not recorded in the Medical Birth Register *(covering 97-99% of all children born in Sweden, depending on the year of birth ) [reference to* [*https://doi.org/10.1007/s10654-022-00947-5*](https://doi.org/10.1007/s10654-022-00947-5) *]*, children who resided less than 5 years in Sweden, children who were adopted, non-singletons and children with missing covariate information (Figure 1A).”

4) **P5, L107. Would this condition typically be diagnosed soon after onset? e.g. is it symptomatic or part of normal screening?**

**Authors’ response:** It is not a condition that is universally screened for during pregnancy. Typically, women report itching as a symptom while pregnant. Abnormalities in bile acid concentrations and liver function tests usually emerge days or weeks later, leading to a diagnosis of ICP. Therefore, even in a clinical setting, the diagnosis of ICP may be delayed after the onset of itching symptoms.

We agree that this clarification may be important for readers who are not familiar with the clinical routines of ICP diagnosis. Therefore, we have now provided additional details in the methods section (Page 7-8; line 164-166):

“*ICP is not universally screened for during pregnancy; typically, patients present with symptoms of itching. Abnormalities in bile acid concentrations and liver function tests usually emerge days or weeks later, leading to a diagnosis of ICP*.”

5) **P5, L118. Is atomexetine used for paediatric cases of narcolepsy? i.e. would all dispensing of this drug be strictly for ADHD?**

**Authors’ response:** Atomoxetine may be used to treat daytime sleepiness in narcolepsy. However, we consider it highly improbable that we have identified any such patients. This is especially evident given that, between 2005 and 2017 in Sweden, only 74 individuals (including adults) with narcolepsy initiated atomoxetine (as reported in <https://onlinelibrary.wiley.com/doi/full/10.1111/ane.13532>). It is much more frequently used in ADHD.

6) **P6, L137. Were robust standard errors used to account for multiple children born to the same mother?**

**Authors’ response:** We appreciate this comment. Indeed, all standard errors were computing using the robust (sandwich) method, to account for multiple children born to the same mother. We have now clarified this in the statistical analysis section of the methods (page 9; line 206-207):

“[…we use logistic regression models with robust standard errors (*to account for multiple children being born to the same mother*).”

Upon reviewing the text in the manuscript, we noticed that we missed including the same statement for the conditional logistic regression. Although we stated in the figure legend that all standard errors were computed using the robust (sandwich) method, we have now corrected this omission (page 10; line 216).

7) **P6, L139. The second model uses a subset of the potential confounders detailed in the previous section. How were these particular covariates selected for the second model?**

**Authors’ response:** To enhance clarity in the covariates section, we have revised it to read:

"We collected a series of *potential confounding variables (factors theorized or confirmed to have a causal impact on both the exposure and the outcome)* from the Medical Birth Register..."

To aid readers, we now also include references for each of the chosen confounders (page 8-9; line 184-191).

We are not entirely certain what the reviewer is referring to regarding omissions in the second model. Several factors mentioned in the relevant paragraph cannot act as confounders, as they must occur after ICP and thus could function as potential mediators or colliders (e.g., fetal characteristics), which should not be adjusted for. We have now clarified this (Page 9; line 192-194):

”[We also collected information on pregnancy and fetal characteristics (when available)], *which we consider to occur concurrently with or after onset of ICP and therefore consider as potential mediators of the relationships examined in this study: …*”

8) **P6, L165. Missing-as-indicator assumes no correlation between the exposure and the missing variable - a more robust option here would be to repeat the analysis with something like IPW or MI that is robust for data that is missing-at-random (i.e. conditional on the observed data).**

**Authors’ response:** We appreciate the opportunity to clarify this point. It's important to note that neither multiple imputation nor other strategies are robust to missing-at-random (MAR) data; they *assume* that the data is MAR. In cases where data is missing-not-at-random (MNAR), complete case analysis is generally considered the least biased alternative, as multiple imputation can introduce bias when data is MNAR.

However, because the true missing pattern is typically not ascertainable from data and we can only speculate about the true missing mechanism, we have also repeated out analysis using ii) performing complete case analysis, ii) treating those with missing data as a separate category ('missing-as-indicator’), iii) performing multiple imputation on categorical BMI (across 20 datasets with multinomial imputation and the exposure, outcome and covariates as auxiliary variables), or iv) using inverse-probability weights to correct for differential missing in BMI (see revised S5 Table). We briefly discuss the findings of this analysis in the manuscript (page 14-15; line 333-341):

“After further adjustment for maternal BMI (irrespective of via complete case analysis, ‘missing-as-indicator’, multiple imputation or inverse-probability weights), slight attenuations were observed in the associations between maternal ICP diagnosed before 28 weeks and between 28-36 weeks of gestation, and offspring NDCs. Similarly, the association between ICP diagnosed before 28 weeks of gestation and offspring intellectual disability was not statistically significant (adjusted OR_complete case analysis_ 1.88, 95% CI 0.69-5.08; adjusted OR_missing-as-indicator_ 1.81, 95% CI 0.74-4.41); adjusted OR_inverse-probability weights_ 1.84, 95% CI 0.51-6.66; adjusted OR_multiple imputation_ 1.82, 95% CI 0.75-4.44) (S5 Table).”

9) **Figure 1. In D), what method was used to create the smoothed incidence curves over age?**

**Authors’ response:** We are thankful for bringing this to our attention. We manually calculated the incidence over age for each birth cohort which gave us the following figure (i.e., the number of incident ‘cases’ per year of age / size of the cohort, for each birth cohort):

For illustration purposes we employ a median-spline with 50 cross-median knots on the aggregate incidence to smooth out the figure (as reported in manuscript):

We have now clarified this in the legend of the figure (page 24; line 586-587).

**Reviewer #3:**

**This is a nice paper. Original research.  No suggestions for improvement.**

**I recommend acceptance.**

**Jim Thornton**

**Nottingham. 31 July 2023**

**Authors’ response:** We appreciate your acknowledgment of this manuscript.

**Reviewer #4:**

**I am asked to comment on revision 1 of this article.**

1) **My main concern is the correction for the main confounder gestational age. As ICP results more often in preterm birth (as the authors acknowledge) this should be controlled for.**

**Authors’ response:** We appreciate this important comment. As we've explained in our responses to other reviewers and the editor (see response to Academic Editor, Comment 1, and Reviewer 1, Comment 3 above), we do not concur that it is appropriate to adjust for gestational age, as it invariably occurs after the onset of ICP. Consequently, it cannot confound the effect; it might, however, act as a mediator. We have now included a directed acyclic graph showing this (revised supplement: S5 Figure).

Nonetheless, we have now performed a complementary analysis we have conducted additional analyses where we: 1) adjusted for gestational age in weeks as a linear term, 2) adjusted for categories of gestational weeks, and 3) adjusted for gestational age using restricted cubic splines to model it in a non-linear fashion. We find that that our estimates are slightly attenuated, but that the qualitative conclusion is the same; early-ICP is associated with offspring neurodevelopmental conditions.

2) **The authors write "and our main model, where we further adjust for birth month, birth order, maternal age, maternal country of birth and maternal psychiatric history and the highest parental education level." Gestational age is not mentioned here. Gestational age is reported as <37, 37-42 and >42 weeks.**

**Authors’ response:** We refer to our above reply (Reviewer #4 Comment 1: Editor’s Comment 1 and Reviewer #1 Comment 3).

3) **They then provide data stratified for gestational age at diagnosis, by reporting them in any, and diagnosis <28, 28-37 and > 37. The stronger effect after the earlier diagnosis is to be expected as these pregnancies will end earlier.**

**Authors’ response:** We agree with you that those diagnosed with earlier-onset ICP will likely end their pregnancies earlier. Indeed, it appears likely that the mediating path explaining why early-onset ICP is associated with offspring neurodevelopmental conditions is earlier delivery. We recognize that this concern has been raised by both the editor and other reviewers. We refer to our above reply (Editor’s Comment 1 and Reviewer #1 Comment 3).

4) **I am afraid the correction for gestational age (if it already takes place?) in categories <37, 37-42 and >42 weeks is insufficient as within these categories the GA of the ICP group will be younger.**

**Authors’ response:** A categorical parameterization of gestational age may indeed obscure patterns important for pregnancies affected by ICP. We have now provided additional analysis where we: 1) adjusted for gestational age in weeks as a linear term, 2) adjusted for categories of gestational weeks, and 3) adjusted for gestational age using restricted cubic splines to model it in a non-linear fashion. This did not change our conclusions.

5) **After these analyses I am happy to review again, but for me at the moment this difference in gestational age might be the main explanation/confounder for what we see here.**
   a) **Authors’ response:** Thank you so much for these important remarks and comments. We hope that you feel that we have rigorously addressed the issue of gestational age, both through causal reasoning and formal adjustment.
