# Supplementary material for: Maternal intrahepatic cholestasis of pregnancy and neurodevelopmental conditions in offspring: A population-based cohort study of 2 million Swedish children
Source: PLoS Med. 2024 Jan 16;21(1):e1004331. doi: 10.1371/journal.pmed.1004331 (PMC10790993; doi:10.1371/journal.pmed.1004331)
Supplement: Attachment — Submitted filename: Editorial comments.docx [file pmed.1004331.s014.docx]

Dear Editors and Reviewers,

We would like to express our sincere gratitude for taking the time to conduct a thorough review of our work. We believe that your insights have significantly enhanced the quality of this manuscript.

Thank you once again for your valuable feedback.

**Editor’s comments**

1. **After discussion among the editorial team and with the Academic Editor, and in line with Reviewer 1, we request that S6 Table (adjusting for gestational age) is incorporated into the main paper.**

***Response:***

We have moved S6 Table to the main body of the paper, where it is now designated as Table 3. Furthermore, we transferred the content of the 8th sensitivity analysis (lines 254-259) from the statistical analysis section to the main analysis, now presented in lines 215-220. Additionally, we shifted the results pertaining to the adjustment for gestational age from lines 354-356 to lines 324-329. We also removed the ‘main analysis’ column from the table. In the previous version, we re-reported our main analysis in Table S6 so that readers would not have to go between the main article and the appendix to compare the estimates. However, this point has now become redundant since we moved the entire table to the main article.

2. **To help us extend the reach of your research, please provide any Twitter handle(s) that would be appropriate to tag, including your own, your co-authors', your institution, funder, etc.**

***Response:***

Shuyun Chen (Twitter: @SC10126), Viktor H. Ahlqvist (Twitter: @AhlqvistViktor), Brian K. Lee (Twitter: @brianklee_epi), Cecilia Magnusson (Twitter: @CecMagnusson), & Karolinska Institutet (Twitter: @karolinskainst).

**Abstract:**

3. **Line 63 - In view of reviewer concerns, we suggest revising the first sentence of your Conclusions to “In this study, we observed that exposure to ICP during gestation is associated with an increased likelihood of neurodevelopmental conditions in offspring, particularly in cases of early-onset ICP” - or similar.**

***Response***:

We have now revised the sentence in line 63 to read:

*“In this study, we observed that exposure to ICP during gestation is associated with an increased likelihood of neurodevelopmental conditions in offspring, particularly in cases of early-onset ICP”.*

**Author Summary:**

4. **Line 106 - We suggest revising to** **“Diagnosis of ICP during pregnancy, especially early in pregnancy, is associated with an increased likelihood of neurodevelopmental disorders in the children exposed during gestation” or similar Line 109 - Please revise ‘causal relationship’. We suggest “...it is not possible to determine** **whether ICP is a causative factor in the development of neurodevelopmental conditions in children born to affected mothers” or similar .**

***Response:***

We have now revised the sentence in line 106 to read:

*“Diagnosis of ICP during pregnancy, especially early in pregnancy, is associated with an increased likelihood of neurodevelopmental disorders in the children exposed during gestation”.*

We have now revised the sentence in line 109 to read:

“Because this study is observational, it is not possible to determine *whether ICP is a causative factor in the development of neurodevelopmental conditions in children born to affected mothers”.*

**Introduction:**

5. **Line 133 - please revise to ‘...by examining the association between…’**

***Response:***

We have now revised the sentence in line 133 (now in line 135) to read:

“We addressed this knowledge gap by examining *the association between* ICP *and* offspring neurodevelopmental conditions (i.e., attention-deficit/ hyperactivity disorder [ADHD], autism, and intellectual disability) in a population-based cohort using the Swedish health and administrative registries”.

6. **Line 139 - please revise to ‘differentially affects the likelihood of’**

***Response:***

We have now revised the sentence in line 139 (now in line 141) to read:

“We therefore also investigated whether the gestational week of ICP diagnosis differentially affects *the likelihood of* offspring neurodevelopmental conditions”.

**Methods:**

7. **Please refer to your prospective analysis plan early in the Methods section.**

***Response:***

We have now relocated the first paragraph of the Statistical Analysis section where we describe the analysis plan and post-hoc analysis (lines 200-203) to the beginning of the Methods section (lines 147-150).

**Results:**

8. **Line 265 - please revise to Children born to mothers with ICP**

***Response:***

We have now revised the sentence in line 265 (now in line 269) to read:

“*Children born to mothers with ICP* were more often male, first-born, born in recent years and between January and March; and more often had well-educated, older mothers, born in a Nordic country, with previous psychiatric conditions, with overweight/obesity, and had more obstetric and neonatal complications (i.e., gestational diabetes mellitus, gestational hypertensive conditions, large for gestational age, caesarean section, preterm birth, low Apgar score, neonatal asphyxia related comorbidities, neonatal hypoglycaemia, and neonatal jaundice) (Table 1)”.

9. **Please provide p values alongside 95% CIs where available.**

***Response:***

We have now included all the P-values alongside the 95% confidence intervals (CIs) in the Results section, wherever available. Please see lines 294-323.

10. **Line 309 - please clarify whether this is adjusted OR**

***Response:***

This is an adjusted OR. We have now revised the sentence in line 309 (now in line 314) to read:

“…, the associations with ICP diagnosed before 28 weeks were similar for offspring ADHD (*adjusted* OR 2.46, 95% CI 1.70-3.55) and intellectual disability (*adjusted* OR 2.34, 95% CI 1.10-4.96), …”.

11. **Line 354 - As you have been so transparent about where results are no longer statistically significant, it is appropriate to comment that adjusting for gestational age affects the statistical significance of the association between intellectual disability and ICP diagnosed <28w, and between autism and ICP diagnosed between 28-36 weeks / any diagnoses of ICP.**

***Response:***

We have now revised the sentence in line 354 (now in line 324-329) to read:

“Adjusting for gestational age slightly attenuated the observed associations but did not alter the qualitative conclusion; early ICP was associated with neurodevelopmental conditions irrespective of controlling for gestational age (*Table 3*), albeit with slightly less precision. *For instance, further adjusting for gestational age altered the statistical significance of the association between ICP diagnosed before 28 weeks and intellectual disability, as well as the association between ICP diagnosed between 28-36 weeks and autism (Table 3).*”

We note that the association between any diagnosis of ICP and autism was statistically significant after adjusting for gestational age in some analysis; we have now included p-values to reflect this, also making the presentation style more aligned with the remaining tables in the main article.

12. **Line 356 - Please incorporate S6 Table into the main paper**

***Response:***

We have relocated S6 Table to the main body of the paper, where it is now designated as Table 3.

**Discussion:**

13. **Line 379 - please revise that adjusting for gestational age had only a negligible effect on the estimates. It would be preferable to acknowledge that adjusting for gestational age affected the statistical significance of the association between intellectual disability and ICP diagnosed <28w, and between autism and ICP diagnosed between 28-36 weeks / any diagnoses of ICP; and to reiterate what is demonstrated in Figure S5.**

**Response:**

We have now revised the sentence in line 379 (now in line 388-396) to read:

“Furthermore, excluding premature births or adjusting for gestational age at birth only had a negligible effect on *most of* our estimates, suggesting that preterm birth is unlikely to explain the stronger associations observed with early-onset ICP. *However,* *adjusting for gestational age altered the statistical significance of the association between ICP diagnosed before 28 weeks and intellectual disability, as well as the association between ICP diagnosed between 28-36 weeks and autism.* *It is important to note, however, that adjusting for gestational age might introduce bias into the results, as it can act as either a mediator or a collider (refer to Figure S5). Therefore, caution is necessary when interpreting results that have been adjusted for gestational age*”.

14. **Line 402 - please replace effect with association.**

**Response:**

We have now revised the sentence in line 402 (now in line 417) to read:

“…to clearly differentiate whether this *association* was due to ICP exposure during a critical window in early fetal development…”.

**Comments from Reviewers:**

**Reviewer #1:**

1. **Thank you for inviting me to rereview this manuscript following author comments. I appreciate the depth and approach to address the comments by the authors throughout.**

**The authors have used arguments developed from mediation analysis to justify their approach of not including gestational age at birth within their initial protocol. I do understand that the authors propose that gestational age at birth itself is likely a mediator between the exposure (ICP) and the outcome (NCD). However, the gestation of birth is determined not only by spontaneous labour onset, but also by clinician-initiated birth; including them as a single pathway risks oversimplification of quite different events, with different relationships both to the exposure (ICP) and, potentially, the outcome (although this is more speculative). Particularly for clinician-initiated birth, the opportunity in this approach of analysis would be to identify a modifiable mediator (and potentially give evidence to support or refute early birth for affected patients). Similarly, I dispute that gestational age of birth is always determined after ICP onset - in clinical practice, iatrogenic birth (e.g. by caesarean section) may be planned before ICP diagnosis and irrespective of this (e.g. in patients with other obstetric indications for early birth) - whilst I don't think that this needs including within this study, it reflects the number of assumptions that have to be made within such a study approach, and supports my recommendation of need to adjust for this (which you have done). In reviewing the mediation analysis, I should emphasise that, whilst I am familiar with the approach, I am by no means an expert in this methodology, so my ability to review that aspect was limited. I have based my understanding upon this BMJ paper (Understanding how health interventions or exposures produce their effects using mediation analysis (bmj.com)) - the editors may wish additional expert review from an epidemiologist if they require additional insight upon this methodology.**

**As such, I do really appreciate the inclusion of the new supplementary table including multiple methods of analysis accounting for gestational age at birth (and would prefer it to be included in the main document - I appreciate that will be more of an editorial decision due to this being post hoc, and with the limitation of space). I still interpret the findings with demonstrated increased risk of NCD and specifically ADHD but do not agree that we can conclude increased risk of autism - I appreciate that the authors have modified their wording to reflect this (even if we disagree a little on the interpretation!).**

**As with all good research, I think that new evidence often raises more questions; I appreciate that the use of mediation analysis requires readers to have an understanding of epidemiological approaches with which clinicians providing direct care may be less familiar, and I anticipate that the findings may generate some useful further discussions within the field.**

***Response:*** We appreciate your thorough review and the valuable insights you have provided on our manuscript. In response to your and the editors’ suggestion, we have moved S6 Table to the main body of the paper, where it is now referred to as Table 3. We echo the reviewer's observation that our work raises more questions; "as with all good research" we hope that our work opens new doors and will be followed by a series of studies on this topic.

Furthermore, we have revised our interpretation of the results, adjusting for gestational age based on both your and the editors’ comments (please also refer to the editors’ comment 11). The modified sentence on line 354 (now in line 324-329) now reads:

“Adjusting for gestational age slightly attenuated the observed associations but did not alter the qualitative conclusion; early ICP was associated with neurodevelopmental conditions irrespective of controlling for gestational age (*Table 3*), albeit with slightly less precision. *For instance, further adjusting for gestational age altered the statistical significance of the association between ICP diagnosed before 28 weeks and intellectual disability, as well as the association between ICP diagnosed between 28-36 weeks and autism (Table 3)*.”

**Reviewer #2:**

1. **Thanks for the revised manuscript and responses to my original review.**

**I agree with the authors that gestational age is inappropriate for the main analyses as changes in gestational age are after any impact of cholestasis, so it can't really be considered a confounder. The analysis including gestational age hints that maybe this is a mediator, but I think this is appropriately left in the appendix.**

**The inclusion of the sensitivity analysis of different approaches to missing data strengthens the manuscript - given the results are very similar with MI and IVP weights this indicates that MNAR is a reasonable assumption in this case.**

**One of the reviewers has legitimate concerns around the findings of this study causing distress to parents. This revised manuscript does make the limitations of the study design clear (i.e. observational) as clear as possible.**

***Response:***

We thank you for your insightful review, which has significantly contributed to the refinement of our manuscript. We believe the changes made in response to the reviews have strengthened the overall quality and clarity of our work.

**Reviewer #4:**

1. **Thank you for the response and adjustments. I have no further questions.**

***Response:***

We appreciate your acknowledgment of the adjustments made to the manuscript and thank you for your time and contributions to the review process. Your feedback has been invaluable in enhancing the quality of our work.
